# Amplification Biases and Consistent Recovery of Loci in a Double-Digest RAD-seq Protocol

**DOI:** 10.1371/journal.pone.0106713

**Published:** 2014-09-04

**Authors:** Jeffrey M. DaCosta, Michael D. Sorenson

**Affiliations:** Department of Biology, Boston University, Boston, Massachusetts, United States of America; CNRS UMR7622 & University Paris 6 Pierre-et-Marie-Curie, France

## Abstract

A growing variety of “genotype-by-sequencing” (GBS) methods use restriction enzymes and high throughput DNA sequencing to generate data for a subset of genomic loci, allowing the simultaneous discovery and genotyping of thousands of polymorphisms in a set of multiplexed samples. We evaluated a “double-digest” restriction-site associated DNA sequencing (ddRAD-seq) protocol by 1) comparing results for a zebra finch (*Taeniopygia guttata*) sample with *in* *silico* predictions from the zebra finch reference genome; 2) assessing data quality for a population sample of indigobirds (*Vidua* spp.); and 3) testing for consistent recovery of loci across multiple samples and sequencing runs. Comparison with *in* *silico* predictions revealed that 1) over 90% of predicted, single-copy loci in our targeted size range (178–328 bp) were recovered; 2) short restriction fragments (38–178 bp) were carried through the size selection step and sequenced at appreciable depth, generating unexpected but nonetheless useful data; 3) amplification bias favored shorter, GC-rich fragments, contributing to among locus variation in sequencing depth that was strongly correlated across samples; 4) our use of restriction enzymes with a GC-rich recognition sequence resulted in an up to four-fold overrepresentation of GC-rich portions of the genome; and 5) star activity (i.e., non-specific cutting) resulted in thousands of “extra” loci sequenced at low depth. Results for three species of indigobirds show that a common set of thousands of loci can be consistently recovered across both individual samples and sequencing runs. In a run with 46 samples, we genotyped 5,996 loci in all individuals and 9,833 loci in 42 or more individuals, resulting in <1% missing data for the larger data set. We compare our approach to similar methods and discuss the range of factors (fragment library preparation, natural genetic variation, bioinformatics) influencing the recovery of a consistent set of loci among samples.

## Introduction

A variety of new “genotype by sequencing” (GBS) methods share the common feature of using one or more restriction enzymes to target a subset of genomic loci for high-throughput DNA sequencing, allowing the simultaneous discovery and genotyping of genetic polymorphisms in a set of multiplexed samples [1]. Widely applicable in both model and non-model organisms, these methods generate massive datasets for a range of applications from genetic mapping to population genetics, phylogeography, and molecular systematics [2]–[5]. The methods described to date vary substantially in the number of loci captured for sequencing (Table 1), but all are designed to recover a specific subset of genomic loci in a more or less consistent manner across samples. Few studies, however, have explicitly evaluated performance or the potential biases leading to differential recovery of loci among samples [6], [7].

**Table 1:** Examples of genotype-by-sequencing (GBS) methods using restriction enzymes and high throughput DNA sequencing to select, sequence and genotype a large set of loci across multiple samples.

Method	# Enzymes	Expected # of loci	Further reduction steps	Year	Ref.
Complexity Reduction of PolymorphicSequences					
(CRoPS)	2	45,440	Pre-selective amplification	2007	[10]
Restriction-site AssociatedDNA Sequencing (RAD-seq)	1	*70,569	None	2008	[8]
Modified CRoPS	2	292,165	Pre-selective amplification,size selection	2010	[37]
Multiplex Shotgun Genotyping (MSG)	1	593,397	Size selection	2011	[38]
Genotype-by-sequencing (GBS)	1	219,256	None	2011	[9]
Modified RAD-seq	2	6,258	Size selection	2012	[11]
Modified GBS	2	83,013	Size selection	2012	[15]
Double-digest RAD-seq (ddRAD-seq)	2	**9,277	Size selection	2012	[6]
Modified GBS	2	445,358	None	2012	[12]
Sequence-Based Genotyping (SBG)	2 or 3	676,355	Third enzyme	2012	[13]
Type IIB RAD-seq (2b-RAD-seq)	1	***27,048	Type IIB enzyme	2012	[14]

*Using SbfI enzyme.

**Using “narrow” size range (see [6]).

***Using selective adapters.

To facilitate comparison, the expected number for each method is estimated for the zebra finch genome based on the specific restriction enzymes and other parameters (e.g., size selection) used in each study.

The optimal level of genome reduction varies with the aims and sampling design of each study [1], [6]. Fortunately, many of these new methods are highly flexible, allowing researchers to balance the number of loci sequenced against the number of samples that can be multiplexed and the expected sequencing depth per sample and locus. The primary tool for adjusting the number of loci is choice of restriction enzyme(s) [8]; enzymes with longer recognition sequences (and/or methylation-sensitive enzymes [9]) cut the genome less frequently and therefore produce fewer loci. Using two enzymes combined with size selection further reduces the number of loci, targeting only those portions of the genome with cut sites for the selected enzymes in close proximity (e.g., [6], [10]–[12]). “Double-digest, restriction-site associated DNA sequencing” (ddRAD-seq) [6] also streamlines fragment library preparation in comparison to the original RAD-seq method [8]. Other means to reduce the number of loci include selective pre-amplification [10], the use of a third enzyme leaving “sticky ends” not compatible with adapters [13], and the use of type IIB enzymes with selective adapters [14].

We implemented a ddRAD-seq protocol with the aim of generating robust genotypic data for a relatively small fraction of the genome (several thousand loci), allowing increased multiplexing and reduced per sample costs for analyses of population structure and gene flow. In comparison to other studies using double-digest methods (e.g., [6], [11]–[13], [15]), we chose enzymes that cut less frequently and used a larger fragment size range (see also [11]). To assess the performance of this approach, we compared empirical results for a zebra finch (*Taeniopygia guttata*) sample to predicted loci from an *in* *silico* digest of the zebra finch genome. This allowed us to characterize biases in the recovery of loci in relation to fragment size, base composition, and genome position. We also explore repeatability across runs by testing the effectiveness of the method in recovering a broadly overlapping set of loci in samples from three species of brood parasitic indigobirds (*Vidua* spp.). Finally, we discuss our results in relation to other recent evaluations of bias in GBS methods [6], [7], [16].

## Methods

### Ethics statement

This study was carried out in accordance with recommendations provided in *Guidelines to the Use of Wild Birds in Research, 3^rd^ Ed.* [17]. Fieldwork, sample collection and genetic analyses were approved by Boston University’s Institutional Animal Care and Use Committee (IACUC protocol numbers: 00–026, 06–033, 10–004, 13–010). The zebra finch sample was obtained from a captive colony at Boston University (IACUC protocol number 11–026).

### ddRAD-seq

We developed a ddRAD-seq protocol similar in basic design to those described in recent studies [6], [11]. Briefly, genomic DNA is cut with two enzymes in a single reaction after which barcoded sequencing adapters with overhangs matching the “sticky ends” produced by the respective enzymes are added in a single ligation reaction. Fragments are then size selected, PCR-amplified, quantified and pooled for sequencing. In comparison to the original RAD-seq method [8], ddRAD-seq targets a smaller subset of loci (assuming the same primary restriction enzyme) and also simplifies the library preparation process by eliminating hydroshearing, end repair, adenylation, and one of two ligation reactions. Choice of restriction enzymes combined with selection of a wider or narrower fragment size range allows substantial control over the number of target loci [6]. A “divergent-Y” in the “P2” adapter prevents amplification of fragments produced by adjacent cuts of the enzyme with higher cutting frequency, yielding a fragment library comprising mostly fragments with “P1” and “P2” adapter sequences on either end, and a smaller number of fragments with a P1 adapter on both ends. The latter affect concentration estimates and bind to the flow cell, but do not form clusters during bridge amplification [18].

### Selection of enzymes and fragment size range

The number of ddRAD loci expected for a given pair of enzymes and fragment size range can be estimated given information on genome size and base composition, but more accurate estimates can be generated through an *in* *silico* digest of an appropriate reference genome. We wrote a python script (*Digital_RADs.py*; available at https://github.com/BU-RAD-seq) that returns the number, size distribution, base composition, and sequences of ddRAD loci that should be generated by a given pair of enzymes and reference genome. To illustrate the difference between RAD-seq and ddRAD-seq, the zebra finch genome sequence includes 70,569 SbfI restriction sites, which should generate an expected 141,138 DNA fragments for sequencing (i.e., upstream and downstream from each cut site) when using the original RAD-seq method [8]. Adding a second enzyme allows great flexibility in the number of loci targeted for sequencing; for example, different enzymes with six-base pair recognition sites can yield as few as 178 or as many as 14,925 fragments in the 200–400 bp size range when combined with SbfI in a ddRAD-seq protocol (Table S1 in File S1). Adjusting the size range allows further modification of the expected number of loci. Ideally, the second enzyme should cut more frequently than the first to minimize the number of fragments with P1 adapters on both ends.

### Laboratory protocols

We outline here the ddRAD-seq protocol we have used to process several batches of samples for analyses of the brood parasitic indigobirds (*Vidua* spp.) and their estrildid finch hosts (a detailed protocol is available in Protocol S1). Some of the results reported in this paper used earlier versions of the protocol, and we highlight any pertinent differences below. We extract genomic DNA using the DNeasy Blood & Tissue Kit (Qiagen Inc.) and estimate concentrations with a NanoDrop instrument (Thermo Scientific). Genomic extracts showing evidence of degradation in an agarose test gel are avoided. We then double-digest ∼1.0 µg of DNA with high fidelity versions of the SbfI and EcoRI restriction enzymes (New England Biolabs); when less DNA is available, we have had good success starting with as little as 0.17 µg of genomic DNA. Following digestion, ligation of double-stranded sequencing adapters is completed in the same tube. The P1 adapter includes the Illumina TruSeq forward amplification and sequencing primer sequences, one of 48 unique, six bp barcodes, and a TGCA overhang on the top strand to match the sticky end left by SbfI (Table S2 in File S1). The 48 barcodes were selected from a set of 128 (Table S3 in File S1) that we designed using Hamming codes [19] such that each barcode has exactly 50% GC content, no more than two consecutive identical bases, and a minimum of two differences with every other barcode. The P2 adapter includes the Illumina TruSeq reverse amplification and sequencing primer sequences, a six bp index sequence, and an AATT overhang on the top strand to match the sticky end left by EcoRI. It also incorporates a “divergent-Y” [20] to prevent amplification of fragments with EcoRI cut sites on both ends (Table S2 in File S1). The barcoded (P1) and indexed (P2) adapters can be used in combination to allow for highly multiplexed libraries [6].

Following ligation, individual samples are run on a 2% low-melt agarose gel and DNA in the 300–450 bp size range is excised from the gel. This size range corresponds to genomic fragments of 178–328 bp after excluding adapter sequences. To aid accurate and repeatable size selection, we add internal size standards of exactly 300 and 450 bp in each lane. To compensate for an amplification bias that favors smaller fragments in the downstream PCR ([21], see Results), we cut a tapered slice from the gel, excising the full width of the lane at 450 bp but only half the width at 300 bp. DNA is extracted from gel slices with the MinElute Gel Extraction Kit (Qiagen) following the manufacturer’s protocol except that the agarose is dissolved at room temperature to increase the representation of AT-rich fragments [22] and we use 20 µL of the Qiagen Elution Buffer. Samples are then amplified for 20 PCR cycles using Phusion High-Fidelity PCR Master Mix (Finnzymes) in a 60 µl reaction with 15 µl of template DNA. Amplified DNA fragments are purified with AMPure XP magnetic beads (Agencourt). Fragment library concentrations for each sample are estimated with quantitative PCR (qPCR) using a KAPA Biosystems kit. Individual fragment libraries are then combined in equimolar amounts and sequenced on an Illumina HiSeq 2000 or 2500 machine. Unless otherwise noted, single end raw sequence reads of 100 bp were generated with TruSeq v3 reagents and CASAVA v1.8 software (Illumina, Inc.). We generated single end sequence data to simplify computational processing of the data, to minimize per sample costs for a large population study, and because there is no opportunity to detect PCR duplicates from paired end reads when using two-enzyme methods like ddRAD-seq. De-multiplexed fastq files for all samples described in this study are available in the National Center for Biotechnology Information (NCBI) Short Read Archive (Accession: PRJNA240988).

### Bioinformatics analyses

We used custom Python scripts (available at https://github.com/BU-RAD-seq) in conjunction with other freely available software to process the Illumina sequence reads. Briefly, sequences passing the preliminary Illumina quality filter are parsed into individual sample files based on P1 barcode and P2 index sequences. The barcode is trimmed from each read and replaced with “CC” to reconstruct the 8-base SbfI recognition sequence. Although the first 6 bases of all reads (and 8 bases including the added “CC”) are identical, we include the full 8-base restriction site to improve the performance of subsequent BLAST searches against the reference (zebra finch) genome. In preliminary analyses, we discovered that many sequences represented unexpectedly short restriction fragments and thus extended through the EcoRI site and into the P2 adapter (see Results); thus, we also search for and remove P2 adapter sequences using an alignment-based approach to allow for imperfect matches; we then add a “C” at the end of these trimmed sequences to complete the EcoRI recognition site. Finally, reads with a complete SbfI or EcoRI recognition sequence in the middle of the sequence, representing concatemers of two different restriction fragments, are either removed from the analysis (SbfI) or trimmed accordingly (EcoRI).

To reduce the size of data files for downstream analysis, we condense identical sequences for a given sample into a single data line, retaining the number of identical reads observed and the highest quality score at each position. Retaining the highest quality score is conservative because multiple identical reads, each with a generally small probability of error, increases confidence in the base call beyond that provided by any single read. We then cluster the condensed reads from each sample using the UCLUST method in USEARCH v5 [23]. Low quality reads (average quality score <20) that do not cluster with any other reads from the same individual at a 90% identity threshold are omitted from further analysis.

Next, the condensed and filtered reads for individual samples are concatenated into a single large file, sorted by average quality score (from high to low), and then clustered into putative loci using UCLUST with an identity threshold of 85%. The highest quality sequence from each cluster is mapped to the zebra finch reference genome using BLASTN v2.2.25 [24] with the following settings: evalue = 0.0001, word_size = 11, gapopen = 5, gapextend = 2, penalty = −3, reward = 1, and dust = yes. Clusters with BLAST hits to the same or approximately the same chromosomal position (±50 bp) and with the same orientation (plus or minus strand) are merged; clusters that do not produce a BLAST hit are carried through the remainder of the pipeline as anonymous loci. We then use MUSCLE v3.8.31 [25] to align the sequences in each cluster (i.e., each putative ddRAD locus).

We developed a custom script to process the aligned sequence data and output haplotype/allele counts, SNPs, and binary coding of each unique indel for each sample and locus. Briefly, our script makes several passes through the data for each putative locus to: 1) identify positions with SNPs and/or indels in one or more samples, 2) identify all unique haplotypes (considering polymorphic sites only) and determine the number of reads for each haplotype in each sample, and 3) evaluate the results for each sample in light of Mendelian expectations. For single-copy autosomal loci, we expect the sequences for each homozygous individual to represent a single haplotype (subject to infrequent errors, typically at different positions in different sequence reads), whereas heterozygous individuals should have two predominant haplotypes, ideally appearing at approximately equal frequency. For the analyses presented here, individuals were scored as homozygous if more than 93% of sequence reads for a given locus were consistent with a single haplotype and as heterozygous if the second most frequent haplotype was represented by >29% of reads. If the second most frequent haplotype was represented by 20% to 29% of reads, the genotype for that individual was flagged as a “provisional heterozygote” and was later “passed” as heterozygous only if both haplotypes were present in other individuals in the population. Samples failing this test and other samples with a secondary haplotype representing 7 to 20% of reads were flagged as ambiguous (“Bad Ratio”). Similarly, a putative heterozygote with a third haplotype representing more than 10% of reads was also flagged as ambiguous (“Extra Reads”). Loci for which multiple samples have ambiguous genotypes (Bad Ratios/Extra Reads) often have high sequencing depth and/or multiple BLAST hits and likely represent duplicated or repetitive elements in the genome (see Results). For loci with segregating polymorphisms, homozygous samples with fewer than five reads were also flagged as ambiguous (“Low Depth”). Finally, if the average quality-score for a variable position across all reads at a locus dropped below 25, we truncated the locus at that position before scoring genotypes.

### Comparison of zebra finch sample to reference genome

To assess the recovery of predicted ddRAD loci, we compared *in* *silico* and empirical results for a single zebra finch sample. We searched the zebra finch genome for all predicted ddRAD loci in the 32–700 bp size range (inclusive of the SbfI and EcoRI restriction sites) and recorded the sequence and base composition for each locus. We then used the first 100 bp of each predicted locus (corresponding to the read length), or the entire sequence for loci less than 100 bp, in BLAST searches against the zebra finch genome. Loci with a single, high quality BLAST hit matching the original location of the predicted tag were included in a stringent set of single-copy loci for comparison to empirical results. We processed a zebra finch tissue sample as described in Protocol S1, but with slightly different PCR conditions (1 µl of each 10 µM primer, 10 µl of template DNA, and 26 PCR cycles), gel purification of the PCR product rather than bead cleanup, and TruSeq v2 reagents. We clustered the empirical sequence reads with the database of predicted single-copy loci using UCLUST, allowing us to determine the number of reads for each predicted locus.

### Consistency among samples and runs for a population sample

We tested the consistency of our ddRAD-seq method by assessing the extent to which a common set of loci was recovered across samples and sequencing runs. We used data for three species of West African indigobirds (*V. camerunensis, V. raricola,* and *V. wilsoni*) collected over the course of six different sequencing runs. These species are closely related and show minimal genetic differentiation at nuclear loci [26], [27]. We first assessed data quality and the number of shared loci among 46 samples in a single sequencing run (“RAD10”) and then used a set of commonly recovered loci to assess consistency among runs, making comparisons to other runs that included 10 or more samples of these same West African species.

We constructed a database of 5,996 putative single copy loci recovered in all 46 RAD10 samples at a depth of five or more sequence reads per sample per locus. Fragment length was estimated from the corresponding locus in the zebra finch genome or, in the case of loci <100 bp in length, measured directly from the indigobird sequence data. Note that the location of the nearest EcoRI site to a given SbfI site often differs between indigobirds and zebra finch; thus, we assumed that estimated lengths greater then 328 bp were incorrect, while also recognizing that some estimates within our size range are also incorrect. We then used USEARCH to cluster the reads for 10 indigobird samples from each of five other runs (RAD5, 6, 14, 16, 18) with the database of RAD10 loci and determined the number of reads representing each locus in each sample. RAD5 fragment libraries were prepared with the same laboratory protocol used for the zebra finch sample (see above). For RAD6, we used a smaller quantity of genomic DNA (∼0.1 µg), pooled batches of 12 samples after ligation of adapters and before the size selection step, and TruSeq v2 reagents. Individual fragment libraries were prepared for the RAD14, 16, and 18 runs following the same protocol used for RAD10.

## Results

### Comparison of empirical zebra finch data to predictions from the reference genome

We analyzed 747,650 reads assigned to an individual zebra finch. Processing these reads through our computational pipeline generated 17,144 “clusters” or putative loci, of which 9,439 were represented by five or more reads. The consensus sequence for most clusters with fewer than 500 reads generated a single BLAST hit or a “best” hit along with other poorer matches (Figure S1 in File S1). In contrast, clusters with sequencing depth of 500 or more reads (*n* = 53, maximum depth = 7,626 reads) typically generated multiple BLAST hits (Figure S1 in File S1) and undoubtedly represent repetitive elements in the genome. Considering the 9,386 putative loci with depths of 5–500, at least 4,295 were heterozygous, with 2 distinct haplotypes, each representing 30–70% of the reads for that locus.

**Figure 1:**
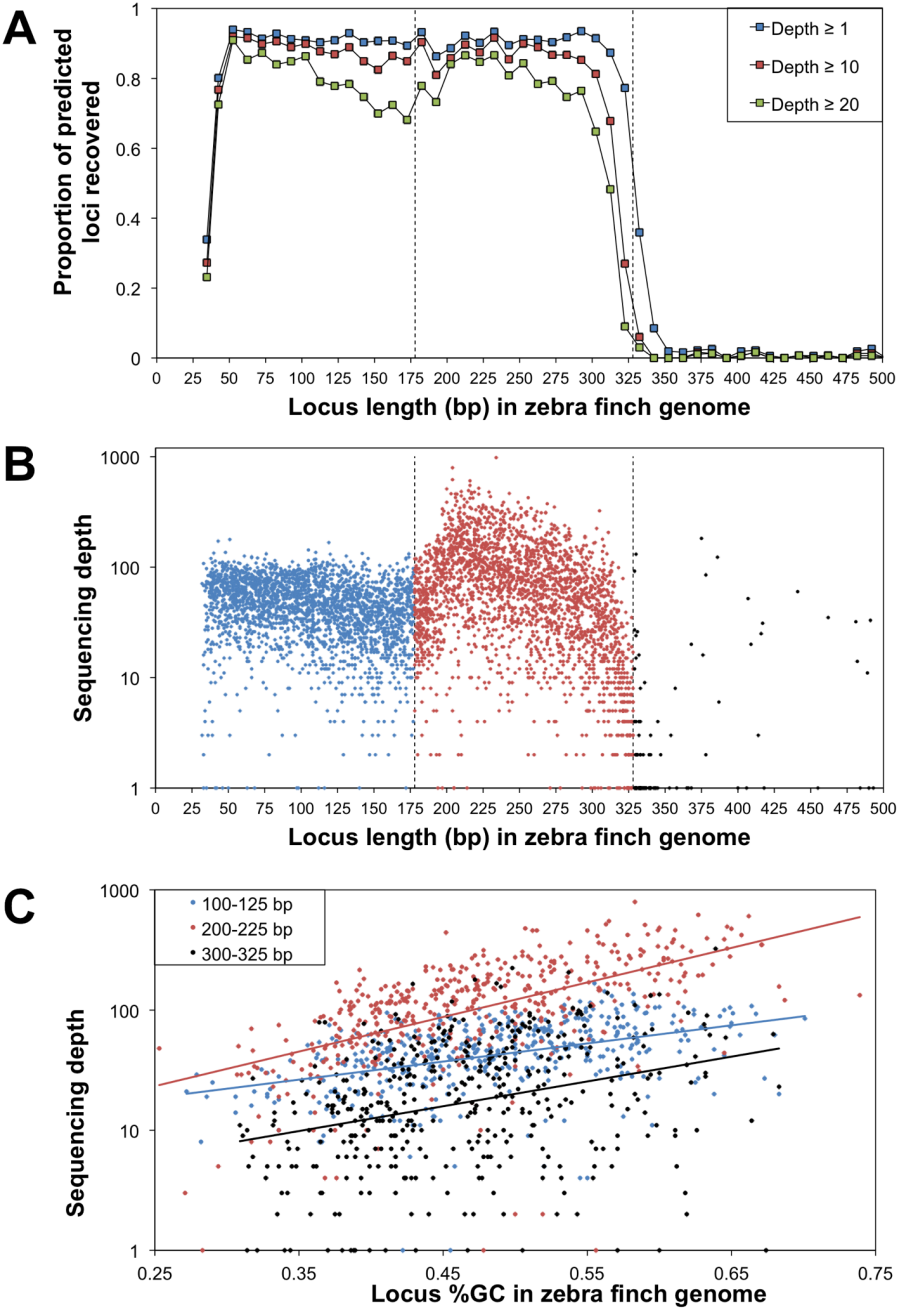
Recovery and sequencing depth for predicted, single-copy ddRAD loci in the empirical zebra finch data. (A) Proportion of predicted loci recovered at three different minimum depth thresholds as a function of predicted fragment length. Each data point represents the proportion of ∼140–220 predicted loci recovered in a given 10 bp size range. Dashed vertical lines represent the upper and lower bounds of the size range isolated from the agarose gel. (B) Sequencing depth for recovered (depth ≥1), single-copy loci in the 32–500 bp size range (includes 5,232 of 5,783 predicted loci in the 38–328 bp size range). (C) The relationship between GC content and sequencing depth for zebra finch ddRAD loci. Data are shown for predicted, single-copy loci recovered at a depth ≥1 in three selected subsets of the overall size range (*n* = 502, 466, and 445 loci in the 100–125, 200–225, and 300–325 bp size ranges, respectively). The predicted length and GC content of each locus are based on the full-length fragment in the reference genome, inclusive of the SbfI and EcoRI restriction sites on either end. Note that the y-axis is on a logarithmic scale in (B) and (C).

Consistent with expectations, recovery of predicted, single-copy ddRAD loci within our targeted size range was generally high, although sequencing depth and the proportion of loci recovered decreased toward the upper limit of the size range before dropping to nearly zero for fragment lengths above the selected size range (Figure 1A). Surprisingly, we also recovered a high proportion of loci in the ∼38–178 bp size range, with a tiny fraction of sequences ranging all the way down to the minimum possible length of 10 bp (13 bp with the restriction sites reconstructed), comprising adjacent SbfI and EcoRI restriction sites overlapping by one base. Considering a stringent set of predicted single copy loci (see Methods), the empirical data included at least one sequence read for 5,232 (90.5%) of 5,783 predicted loci in the 38–328 bp size range, and 5,078 of these loci (87.8% of the predicted loci) were represented by at least five reads. A small number of recovered loci with predicted lengths longer than 328 bp are presumably due to restriction site polymorphisms, indels generating fragment length differences between the reference genome and our individual sample, or a low level of star activity (see below).

Sequencing depth per locus within the recovered size range varied with both fragment length (Figure 1B) and base composition (Figure 1C). Sequencing depth was highest for loci that were ∼200 bp in length and was negatively correlated with length in the ∼200–328 bp range (Figure 1B). Within this range, both fragment length and base composition explained a significant portion of the variation among loci in sequencing depth (multiple linear regression, *R* ^2^ = 0.43, *p*<0.0001; partial *R* ^2^ for length = 0.20, *p*<0.0001; partial *R* ^2^ for GC-content = 0.23, *p*<0.0001). By making a tapered cut of the gel slice during size selection, we reduced the relative representation of smaller fragments, generating a positive correlation between depth and fragment length in the ∼178–200 bp range (Figure 1B, Figure S2 in File S1). Sequencing depth was not strongly related to fragment length for loci smaller than the selected size range (i.e., ∼38–178 bp). For all loci, sequencing depth was positively correlated with GC content; this relationship was stronger within the selected size range than it was for shorter loci (Figure 1C).

The base composition of recovered ddRAD loci was not representative of the entire genome. While the overall base composition of the zebra finch genome is 41.4% GC, the base composition of the 5,783 predicted, single-copy ddRAD loci in the 38–328 bp size range is 48.1% GC, excluding the restriction sites. Likewise, average base composition for the subset of loci with one blast hit and 5–500 reads was also 48.1% GC. This bias towards GC-rich regions is expected given that the SbfI recognition sequence (CCTGCAGG) is 75% GC and the combined base composition of the SbfI and EcoRI recognition sites is 57% GC.

**Figure 2:**
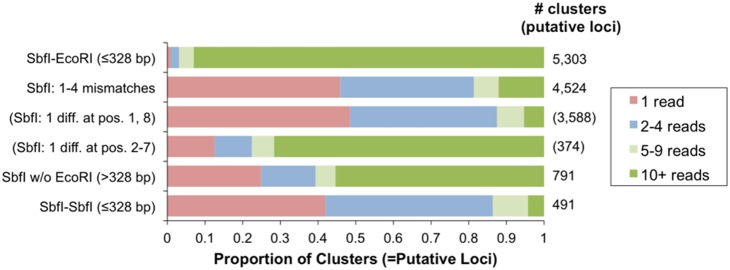
Sequencing depth for single copy ddRAD loci in relation to the corresponding sequence in the zebra finch reference genome. Categories from top to bottom include: loci mapping as expected to predicted SbfI-EcoRI restriction fragments≤328 bp in length; all loci beginning at a genomic location similar but not identical to the canonical SbfI recognition sequence (1–4 mismatches); subset of loci with one mismatch in position 1 or 8 of the SbfI recognition sequence; subset of loci with one mismatch in positions 2 through 7 of the SbfI recognition sequence; loci mapping to a genomic SbfI site without an EcoRI site within 328 bp; and loci mapping to a predicted SbfI-SbfI restriction fragment less than 328 bp in length.

As noted above, we recovered 17,144 putative loci from our zebra finch sample, thousands more than the 10,120 loci (38–328 bp size range) predicted by an *in* *silico* digest of the reference genome (note that most of the above analyses were based on a smaller, stringent subset of predicted single copy loci). Mapping our empirical data to the genome identifies two processes that increase the number of loci represented in the fragment library: star activity (i.e., non-specific cutting by the restriction enzymes) and ligation of two or more restriction fragments during fragment library preparation (i.e., concatemerization). Focusing on 11,309 empirical loci that produced a single, high-quality BLAST hit reveals four distinct categories:

1. 5,303 loci (46.9%) map, as expected, to a predicted SbfI-EcoRI fragment less than 328 bp in length; these loci were generally recovered at relatively high depth (median depth = 47; 93.0% had ≥10 reads) (Figure 2).
2. 4,524 loci (40.0%) map to a genomic location with an 8 bp sequence similar but not identical to the canonical SbfI recognition site (1 to 4 mismatches). Most loci in this category start at a genomic location that differs from the SbfI recognition site by a single mismatch in either the first or last position (90.6% of 3,962 loci with one mismatch), and most of these loci were recovered at low depth (median depth = 2; 94.7% had ≤9 reads), consistent with a low level of non-specific enzyme activity at such sites. In contrast, loci differing by a single mismatch in positions 2 through 7 accounted for only 374 (9.4%) of these loci but were typically recovered at much higher depth (median depth = 24; 71.7% of loci had ≥10 reads), suggesting that most loci in this latter category represent SbfI-EcoRI fragments generated by restriction site polymorphisms between the reference genome and our zebra finch sample.
3. 791 loci (7.0%) map to an SbfI site without a nearby EcoRI site. Sequencing depth for these loci was variable (Figure 2); we suggest that most of the low-depth loci in this category are generated by non-specific activity of EcoRI at non-canonical EcoRI sites near SbfI sites, whereas most loci recovered at higher depth represent EcoRI restriction site polymorphisms between the reference genome and our sample.
4. 491 loci (4.3%) map to a predicted SbfI-SbfI restriction fragment less than 328 bp in length; most of these loci were recovered at low depth (Figure 2). In preliminary analyses, we identified a small number of sequences representing the ligation of SbfI-SbfI restriction fragments to SbfI-EcoRI fragments, generating chimeras with the necessary sequencing adapters on either end. Thus, our computational pipeline checks for and discards sequences that include a complete SbfI restriction site, but chimeras with a reconstituted SbfI site beyond the read length are not detected and are thus retained in the final data set. A few of these loci (*n* = 21, 4.3%) were recovered at higher depth (≥10 reads) and may represent “intended” SbfI-EcoRI fragments, reflecting polymorphisms responsible for gain of EcoRI or loss of SbfI sites in the downstream sequence.

### ddRAD-seq results for a population sample

We pooled ddRAD-seq libraries for 46 indigobird samples (RAD10), representing three species from Cameroon, and generated ∼30.8 M reads in a single lane of an Illumina HiSeq 2000 flow cell; this was somewhat fewer reads than anticipated due to an issue with library quantification. We achieved approximately equal representation across individual samples (mean ± sd: 562 K±55 K assigned sequence reads; range: 476 K–767 K). Likewise, a broadly overlapping set of loci was recovered across all 46 samples; our computational pipeline yielded 5,996 putative single copy loci that were recovered with ≥5 reads and successfully genotyped in all 46 samples, including 2,109 invariant loci and 3,887 loci with one or more polymorphisms (SNPs and/or indels). Most of these loci (91.1%) generated a BLAST hit to the zebra finch reference genome (median e-value of 7E-29). Of the remaining 531 loci, 392 produced a BLAST hit when compared to the NCBI reference genomic sequences database. All of these hits were to avian taxa, and another passeriform was the closest match in almost all cases (99.0%). Sequence data (≥1 read per sample per locus) were obtained for at least 90% of individuals (42 of 46) for an additional 1,283 invariant and 2,554 variable loci. These totals exclude 548 clusters/putative loci with data for ≥42 samples, but also three or more “flagged” genotypes (“bad ratio”, “extra reads”); many of these clusters include sequences from loci with two or more similar copies in the genome. We used several additional criteria to screen for and exclude duplicated loci, including: 1) unusually high average read depth; 2) a strong excess of heterozygotes as compared to Hardy-Weinberg expectations; 3) highly divergent alleles; and/or 4) consistently higher read depth for heterozygotes than homozygotes, a pattern generated when a second, similar locus was recovered in only a subset of samples. For the larger set of 9,833 loci, less than 0.2% of genotypes were flagged, whereas data were missing for ∼1% of genotypes and ∼10.8% of genotypes had low sequencing depth (<5 reads) (Table 2).

**Table 2:** Genotyping success for 9,833 loci recovered in at least 42 of 46 indigobird samples in the “RAD10” run.

	Constant Loci		Variable Loci		
	46 samples	42–46 samples*	46 samples	42–46 samples*	Total
Number of loci	2,109	1,283	3,887	2,554	9,833
Median read depth per sample	45	10	61	8	25
Total number of genotypes	97,014	59,018	178,802	117,484	452,318
Number with “low depth”	–	14,850	–	33,920	48,770 (10.8%)
Number of missing genotypes	–	1,200	–	3,086	4,286 (0.9%)
Number with “bad ratio”	–	–	–	811	811 (0.2%)
Number with “extra reads”	–	–	–	69	69 (0.02%)
Total missing or “flagged”	–	1,200	–	3,966	5,166 (1.1%)

See Methods for more information on categories of “flagged” genotypes.

*Includes loci with ≥ one read for at least 42 of 46 samples and no more than 2 “flagged” genotypes.

**Figure 3:**
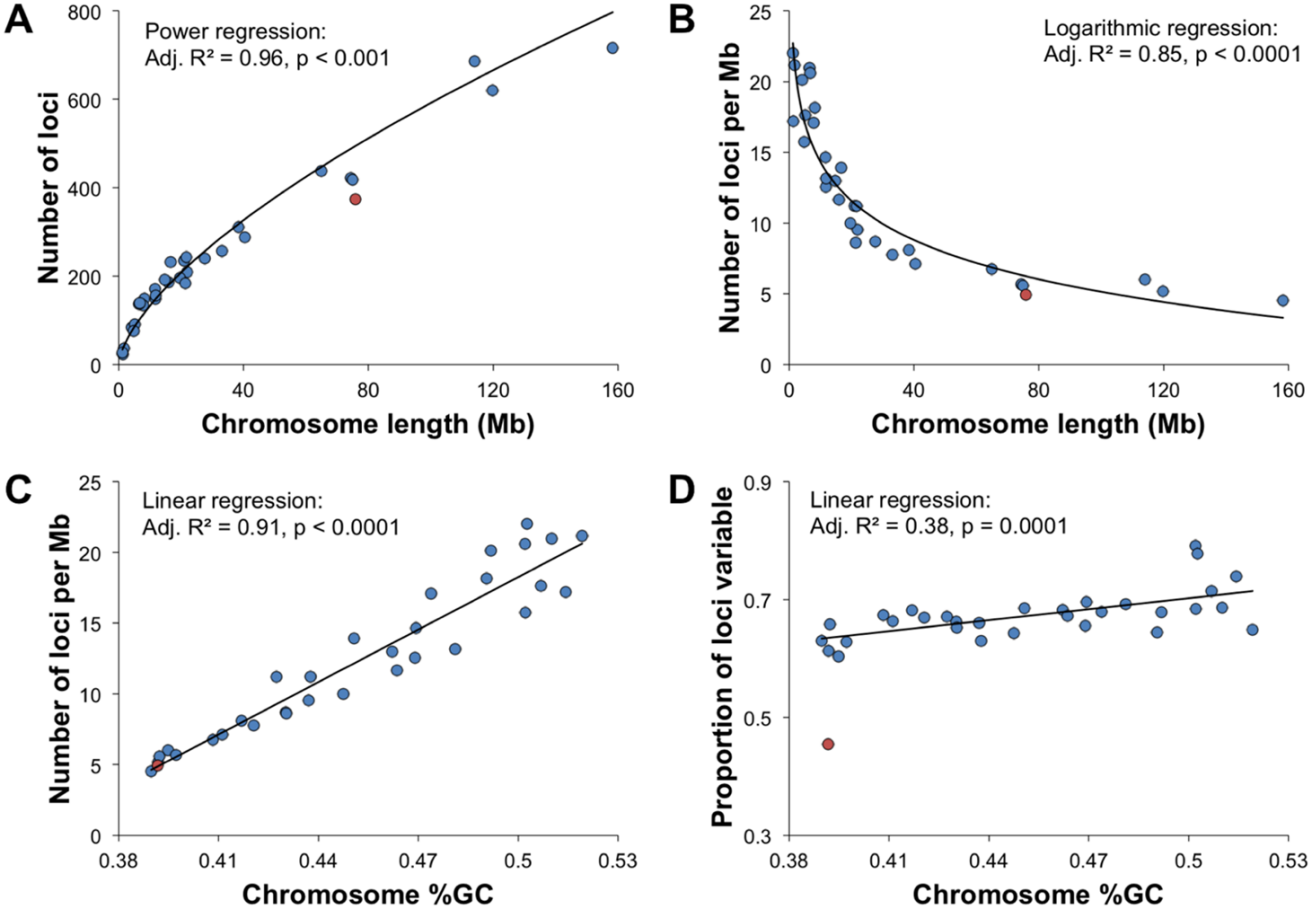
Genomic distribution of indigobird ddRAD loci based on BLAST results against the zebra finch reference genome. Includes 7,819 loci that had one or a “best” BLAST hit and were genotyped in at least 42 of 46 samples (*n* = 5,045 variable loci, *n* = 2,774 constant loci). Data for the “chrUn” contig and small contigs with no BLAST hits (e.g., “chr16”, “LG2”, “LG5”) are excluded. (A) The number of loci mapped to each chromosome as a function of chromosome length. (B) The density of loci as a function of chromosome length. (C) The density of loci as a function of chromosome GC content. (D) The proportion of loci that was variable as a function of chromosome GC content. The Z-chromosome is indicated by a red point in each panel and was not used in regression analyses.

Consistent with the relationships between fragment length, base composition, and sequencing depth noted above, sequencing depth varied among loci and this variation was strongly correlated among individuals. Locus identity explained a far greater proportion of variation in sequencing depth than sample identity (two-way ANOVA of 2,000 randomly selected loci: partial *η* ^2^ effect sizes of 0.82 and 0.02 for locus and sample, respectively). BLAST results indicate that recovered indigobird loci were broadly scattered across the genome. As would be expected, there was a significant positive correlation between zebra finch chromosome length and number of indigobird loci mapping to each chromosome, but with an up to four-fold over representation of loci on generally smaller, GC-rich chromosomes (Figure 3A–C). The proportion of loci that were polymorphic also increased with GC content (Figure 3D).

We further assessed data quality by examining PCR and/or sequencing error rates as well as the fit of our data to Mendelian and population genetic expectations. To simplify these analyses, we focused on 1,721 loci that were scored as having a single bi-allelic SNP and that were recovered in all 46 samples with no flagged genotypes (results were entirely comparable for loci with a larger number of polymorphisms/alleles, but are more complex to summarize). For reference, these 1,721 loci had median sequencing depth of 58 reads per sample per locus, with heterozygotes having a median of 28 reads per allele. Consistent with low rates of PCR and/or sequencing error, 99.48% of all reads (*n* = 5.2 M) were identical across their full length to one of the two consensus allele sequences at each locus; thus, sequences for all these loci were effectively “replicated” across multiple samples. Of those sequences that did not match perfectly, 96.8% were either singletons (i.e., observed only once in a given sample; 66.4% of mismatched reads) and/or were sequences that differed from one of the consensus allele sequences at a position(s) with quality score <30 (69.7% of mismatched reads), the latter indicating sequencing error rather than PCR error as the predominant source of error. In a few cases (*n* = 14 individual genotypes at 13 loci; 0.018% of all genotypes), an individual had 2 to 9 high quality reads comprising 20–30% of its reads; these likely represent rare polymorphisms that did not meet the initial threshold for identifying a variable site in our genotyping code.

**Figure 4:**
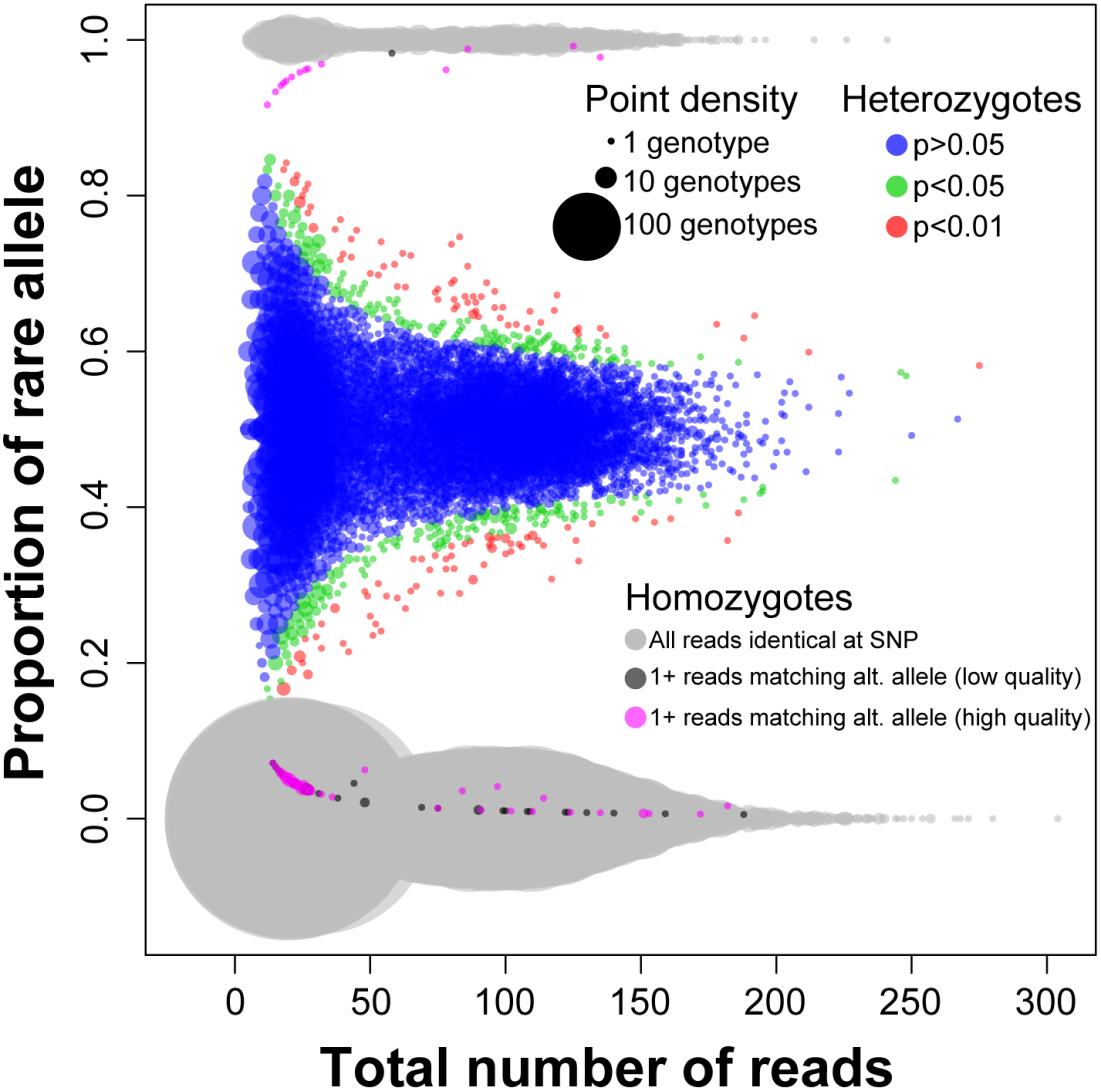
Read counts for indigobird ddRAD loci with a single bi-allelic SNP. Data are shown for 1,721 loci recovered in all 46 indigobirds in the RAD10 sequencing run (*n* = 79,166 genotypes). The area of data points is proportional to the number of individual genotypes at each coordinate. Read counts for heterozygous genotypes (*n* = 11,761) were consistent with random sampling from a binomial distribution with probability 0.5 (i.e., Mendelian expectations), resulting in a strongly trimodal distribution of read counts. Most homozygous genotypes (*n* = 67,405) were based on 100% of reads (light grey) matching one of the two alleles at a given locus; only 64 genotypes (0.09%) scored as homozygous had one or more high quality reads (magenta) matching the alternative allele.

Consistent with Mendelian expectations, read counts for 79,166 genotypes (1,721 loci × 46 individuals) were strongly tri-modal, with homozygotes having either ∼0% or ∼100% of reads matching the rare allele at the SNP position and heterozygotes having reads matching both alleles (Figure 4). Read depths for the two alleles in heterozygotes were consistent with stochastic sampling from the binomial distribution, with reduced variation around the 50/50 expectation as total depth increased. The proportion of heterozygous genotypes deviating from binomial expectations at the 0.05 confidence level (4.6%; 537 of 11,761), including 1.3% (*n* = 151) at the 0.01 confidence level, was approximately what would be expected by chance. Only 64 of 67,045 homozygous genotypes (0.095%) included one or more high quality reads that matched the alternative allele at the SNP position; in cases of low depth (e.g., <20 reads total), there is a small chance that some of these were heterozygotes incorrectly scored as homozygous, but either PCR or sequencing error (including errors in the barcode sequence) might also account for a single mismatched read.

**Figure 5:**
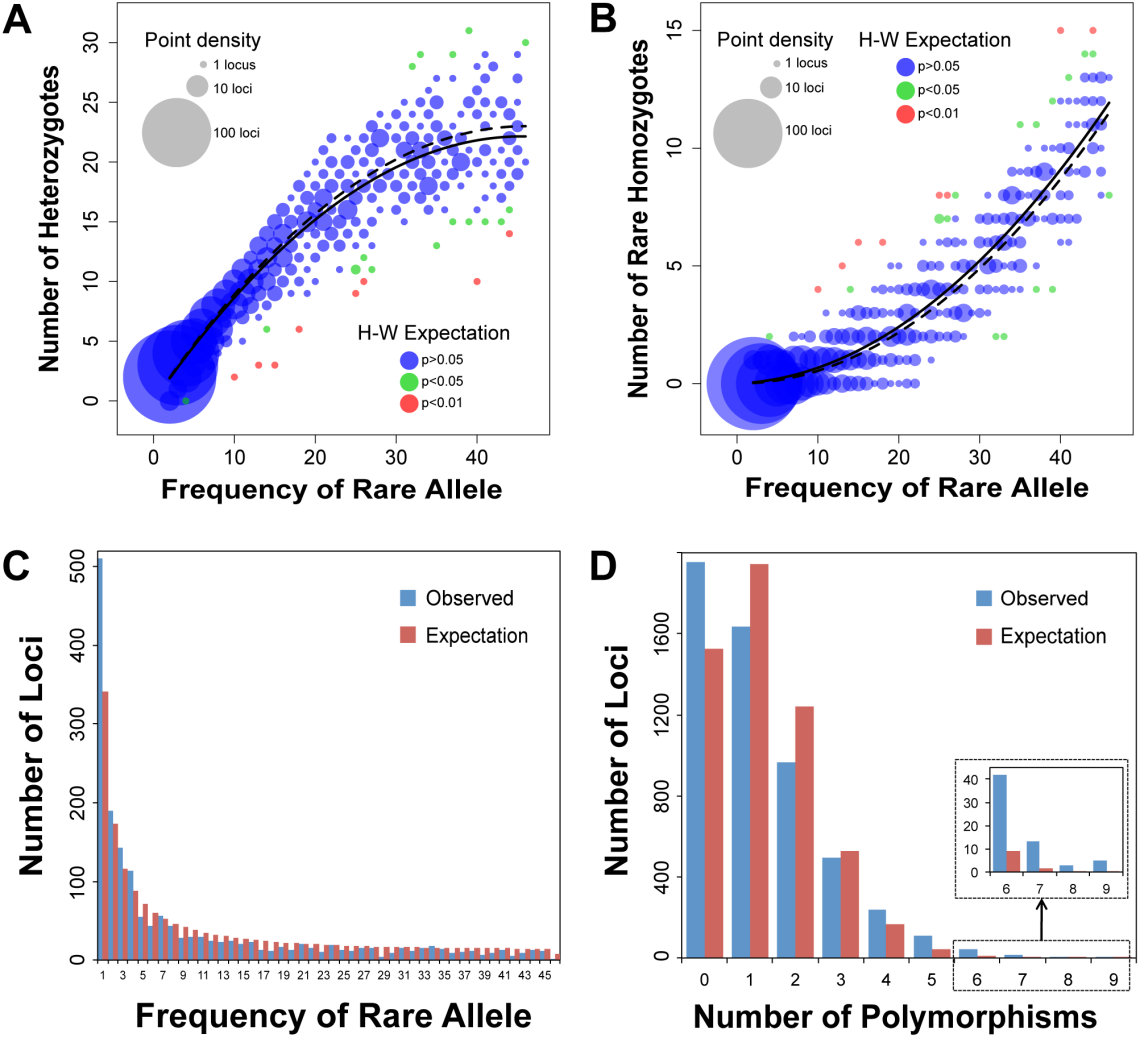
Comparison of indigobird ddRAD results to population genetic expectations. (A) Number of heterozygotes and (B) number of homozygotes for the rare allele versus rare allele frequency for 1,721 loci with a single bi-allelic SNP. The area of data points is proportional to the number of individual genotypes at each coordinate; observed relationship (sold lines), expected (dotted lines). Loci deviating from Hardy-Weinberg expectations are highlighted in green and red. (C) Comparison of the empirical allele frequency distribution for the same 1,721 loci with neutral expectations for a population of constant size. (D) Distribution of polymorphisms among full-length (97 bp) loci genotyped in all 46 samples in RAD10 compared to a Poisson distribution, which assumes equal evolutionary rates across loci.

Genotyping accuracy is also supported by an approximate fit of genotype frequencies to Hardy-Weinberg expectations, with both heterozygotes and homozygotes for the rare allele increasing with allele frequency (Figure 5A–B). A small fraction of loci with a deficiency of heterozygotes can be attributed to combining data from three closely related species with minimal genome-wide differentiation (i.e., the Wahlund effect [28]); nearly all of these loci have *Φ*_st_ values in the right tail of the distribution (range 0.15 to 0.62) as compared to the genome-wide value of *Φ*_st_ = 0.047. Likewise, the distribution of rare allele frequencies at these same loci (i.e., the site frequency distribution) is roughly consistent with neutral expectations for a population of constant size (Figure 5C), albeit with a moderate excess of low frequency alleles, which is consistent with population expansion. Finally, expanding the analysis to all loci recovered in all 46 samples, the observed level of polymorphism (mean = 1.27 polymorphisms per locus) was consistent with previous indigobird studies [26], [27], and the distribution of polymorphisms among loci was approximately Poisson distributed (Figure 5D).

### Consistency among runs in sequenced ddRAD loci

**Figure 6:**
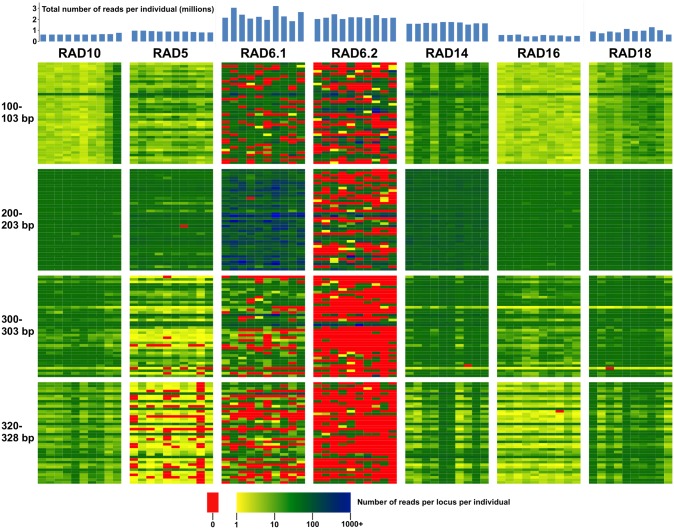
Recovery of indigobird ddRAD loci across individual samples and sequencing runs. Sequencing depth for 160 selected loci (rows), including 40 loci in each of four narrow size ranges, is shown for 10 randomly selected samples (columns) from each of seven pooled libraries. The 160 loci illustrated are a subset of the 5,996 loci recovered in all 46 indigobirds sequenced in the RAD10 sequencing run. Overall sequencing depth for each individual sample is show in the bar graph at top. Sequencing depth for each locus is indicated by color (see scale at bottom of figure), with red indicating no data. See text for more information.

A broadly overlapping set of loci was recovered across most but not all of our sequencing runs (Table 3, Figure 6). This was particularly true for later runs using the same laboratory protocol (i.e., RAD10, 14, 16, 18); using 5,996 “core” loci from RAD10 samples as a reference set, subsequent runs recovered 95.7% (RAD14), 92.9% (RAD16) and 98.0% (RAD18) of these loci at a depth of 5+ reads in all 10 samples analyzed, and individual samples were missing data for a small fraction of loci (Table 3). In these runs, core loci were recovered at a high rate across the selected size range (Figure 6, Figure S3 in File S1), with a strong correlation across runs in per locus sequencing depth (Figure S4 in File S1). Slightly poorer success in RAD16, which had similar per sample read depth to RAD10, was apparently due to one or more factors influencing quality of the fragment library (Figure S5 in File S1). Our RAD5 run used different PCR parameters (see Methods) and gel-based rather than bead-based purification of PCR products. Despite these differences, the set of core loci was well represented; 88.2% of loci had at least five reads for each of the 10 individuals analyzed in RAD5 (Table 3). Loci with missing data tended to be toward the upper limit of the size range (Figure 6, Figure S3 in File S1). Variation in sequencing depth across samples and within loci was relatively low in all of these runs; the average coefficient of variation in read depth was below 0.3 in each run (Table 3).

**Table 3:** Summary statistics characterizing the performance of multiple sequencing runs in recovering a core set of loci.

	Samples	Depth per sample	Missing loci per sample	CV per locus depth	Loci recovered at
Run	(*N*)	(avg±sd)	(avg±sd)	(avg±sd)	≥5x in all samples
RAD5	10	895 K±51 K	38±35	0.27±0.16	5,289
RAD6 pool 1	10	2,377 K±459 K	732±166	0.71±0.37	2,640
RAD6 pool 2	10	2,170 K±140 K	2,634±80	1.37±0.81	714
RAD14	10	1,651 K±67 K	6±1	0.23±0.13	5,740
RAD16	10	536 K±53 K	46±4	0.26±0.16	5,571
RAD18	10	929 K±194 K	9±16	0.28±0.07	5,879

Analysis based on a set of 5,996 loci genotyped in all 46 samples in RAD10 with per locus sequencing depth ≥5 reads for all 46 samples.

Pooling samples earlier in the library preparation process (e.g., after digestion and ligation of barcoded adapters) offers considerable savings in both the time and costs associated with preparing samples for ddRAD-seq [6]. We used this approach for our RAD6 run (see Methods). Despite generating many more sequence reads per sample (Table 3), the reference set of loci was recovered much less consistently in RAD6 as compared to other runs. While loci with the highest average depths in RAD10 were generally recovered in most or all RAD6 samples, the RAD6 run showed a pattern of seemingly random “dropout” of loci throughout the entire size range (Figure 4, Figure S4 in File S1), resulting in much greater within locus variability in read depth among samples (Table 3). Results also differed between two batches of pooled samples in RAD6: 44.0% of the RAD10 reference loci were recovered with ≥5 reads in all 10 samples in RAD6-pool-1, whereas only 11.9% were consistently recovered in RAD6-pool-2. Recovered loci often had many more reads (e.g., 1,000+) than necessary to determine genotypes. These results illustrate that high per sample sequencing depth does not necessarily translate into good representation across loci or consistent recovery of loci among samples.

## Discussion

A variety of conceptually similar GBS methods have been introduced in recent years, providing new opportunities and increased power for addressing a wide range of questions in molecular ecology, evolutionary biology and related fields. Designed to sample a specific subset of the genome across multiple samples, the utility of these methods depends on consistent recovery of loci across batches of samples, but there has been limited evaluation of this key aspect of performance (but see [6], [7], [16]). Potential pitfalls and biases associated with laboratory protocols, natural genetic variation, and computational processing of the sequence data all may affect the degree to which a common set of homologous loci is recovered across samples. We discuss below the range of factors influencing performance and how the details of different ddRAD-seq protocols may influence results.

### Recovery and amplification biases associated with fragment library preparation

While the ideal GBS method would yield uniform sequencing depth across all recovered loci, both the original RAD-seq method and ddRAD-seq generate substantial variation, albeit for different reasons. In the original method, there is a strong positive relationship between restriction fragment length and sequencing depth, apparently due to the poor efficiency of hydro-shearing shorter fragments [7]. This bias is not relevant to ddRAD-seq, which replaces hydro-shearing with a second restriction enzyme that determines final fragment length; thus, we found a negative relationship between fragment length and sequencing depth within our selected size range (Figure 1B), presumably due to amplification bias in favor of shorter molecules [21]. We also detected a significant bias in favor of loci with higher GC content (Figure 1C), an effect also observed in the original RAD-seq method with an increasing number of PCR cycles [7].

Our ddRAD-seq protocol is generally similar to that described by Peterson *et al.* [6], but we used enzymes that cut less frequently combined with a larger fragment size range to recover a comparable number of loci (Table 4). A narrower size range is preferable for Illumina sequencing [22] and reduces the potential for amplification bias but it puts a premium on precise and consistent size selection. Indeed, Peterson *et al*. [6] focused on this issue as a key to good performance and demonstrated the advantages of automated size selection using a Pippin Prep instrument (Sage Science). We obtained excellent results using a standard agarose gel and wider size range combined with enzymes that cut less frequently. Under these conditions, slight error in the selected size range affects a smaller proportion of the targeted loci. Except for our RAD5 run, which used gel-based rather than bead-based purification of PCR products, we achieved consistent recovery of loci all the way to the upper limit of our selected size range (Figure 6, Figure S4 in File S1).

**Table 4:** Number of predicted ddRAD loci in the zebra finch genome for alternative restriction enzymes and fragment size ranges.

	Selected Size Range		
	This study:	Peterson *et al.*:	
	38–328 bp	230±24 bp	230±36 bp
Enzyme Pair	(300 bp)	(48 bp)	(72 bp)
SbfI-EcoRI (this study)	10,120	1,751	2,613
EcoRI-MspI (Peterson)	66,672	9,277	14,115

All reported values are based on *in* *silico* digest of the zebra finch reference genome. Table compares our “realized” size range (with “small fragment carryover”, see text) with two size selection options employed by Peterson *et al*. [6].

An unexpected consequence of size selection in a standard agarose gel was the recovery of fragments shorter than the lower limit of our targeted size range (Figure 1A–B, Figure S2 in File S1). This “small fragment carryover,” revealed by directly comparing empirical data with expectations from a reference genome, has not been reported in previous ddRAD-seq studies (e.g., [6], [11]). Because sequences for the shortest fragments (<100 bp) extended into the P2 adapter, searching for P2 adapter sequences and trimming sequences accordingly was a critical step in the initial processing of our data. While the inclusion of short fragments reduces data collection efficiency, the consistency of the effect across samples produced a set of shared loci in the 38–178 bp range, a large fraction of which were variable and genotyped in all samples. While the exact mechanism is uncertain, this effect is apparently substantially reduced by including a bead-based purification step following digestion of the genomic DNA and/or by using a Pippin Prep for size selection (Sage Science, pers. comm.).

Although our realized fragment size range was substantially wider than intended, excessive variation in sequencing depth was likely ameliorated by two factors: 1) for smaller fragments (38–178 bp), we speculate that a positive relationship between “carryover probability” and size was countered by amplification bias favoring short fragments, resulting in relatively constant average sequencing depth across this range (Figure 1B); and 2) making a tapered gel slice during size selection was effective in reducing amplification bias at the lower edge of our selected size range (∼178–200 bp; Figure 1B, Figure S2 in File S1).

As in previous studies [6], [7], we found that recovery/amplification biases affect all samples in a similar manner, resulting in strong covariation among samples in sequencing depth across loci. Peterson *et al*. [6] attributed this effect to a negative correlation between depth and distance of a given locus from the midpoint of the selected size range, presumably due to lower recovery of fragments farther from the midpoint. With direct estimates of fragment length from the zebra finch genome, our results show that amplification bias in relation to both fragment length and GC-content also contributes to correlated variation in depth across loci. Even from 206 bp to 254 bp, which corresponds to the “narrow” range used by Peterson *et al.* (but is well within the limits of our selected size range), we observe a 25% reduction in median per locus sequencing depth in our zebra finch test sample. The relatively large number of PCR cycles we used may exacerbate amplification biases in relation to both fragment size and GC content, but the consistency of this effect contributes to the recovery of a “core set” of high-depth loci in all or almost all samples, which can be viewed as an advantage of ddRAD-seq (see also [6]). Note that PCR ramp time is another factor that may have a significant impact on amplification biases [29].

Our RAD6 run illustrates an additional important point: inconsistent recovery of ddRAD loci across samples may result from laboratory failures unrelated to size selection. Despite generating 2+ million reads per sample, we obtained highly uneven representation of the targeted loci and observed a pattern of seemingly random “dropout” throughout the selected size range (Figure 6). To reduce library preparation time and costs, we pooled the RAD6 samples after ligation and prior to size selection, but we suspect the poor performance of this run was due primarily to reducing the quantity of input genomic DNA per sample (from 1 µg to 100 ng) combined with inefficient recovery during one or more purification steps prior to PCR amplification. Note that a tiny fraction of the genome may be represented in a ddRAD-seq fragment library: for example, ten thousand ∼250 bp loci represent only ∼0.2% of the zebra finch genome. With our protocol, 100 ng of genomic DNA yields only 0.2 ng of ligated fragments in the selected size range, assuming 100% efficiency. Degraded input DNA, inefficient digestion or ligation reactions, and/or poor recovery during size selection may further reduce the quantity of DNA taken into the PCR step. In more recent work (Stryjewski *et al.*, in prep.), we have achieved results comparable to RAD10 and subsequent runs when pooling batches of 12 samples prior to size selection; in these runs, we have digested ∼1.0 µg of genomic DNA per sample and then used qPCR to quantify the concentration of successfully ligated fragments in each sample before pooling equimolar amounts.

A final factor generating inconsistent recovery of loci among samples is restriction enzyme star activity (i.e., cutting at non-canonical recognition sites). By comparing our empirical zebra finch data to the reference genome, we discovered a considerable level of star activity (even when using “high fidelity” versions of the enzymes) concentrated at sites with a mismatch in the first or last base of the recognition sequence (Figure 2). Comparison to another recent study [30] suggests that the specific patterns of non-canonical activity may vary with restriction enzyme and flanking sequence. In our study, star activity generated a large number of “extra” loci sequenced at low depth (typically one or a few reads), and, for the most part, these loci were non-overlapping among samples and inconsequential for downstream analysis. At other loci, however, star activity may result in the recovery (at low depth) of alleles that would otherwise be null alleles (due to restriction site polymorphisms, see below); this presents complications both for the detection of null alleles and for the potential use of presence-absence data in phylogenetic or other analyses.

### Biases and challenges related to natural genetic variation

GBS methods are sometimes portrayed as providing a “random” sample of loci across the genome, but our analysis clearly demonstrates the interaction between choice of restriction enzymes and the number loci recovered from different parts of the genome. While our method, and any other method based on restriction enzymes, broadly samples the genome, it does not randomly sample it. Our use of SbfI, which has a recognition sequence that is 75% GC, as the less frequent cutter resulted in an over-representation of loci with higher average GC content than the genome as a whole and up to four-fold over-representation of loci on the relatively small, GC-rich avian microchromosomes (Figure 3B–C). The higher rate of sequence evolution on these chromosomes [31], [32] also resulted in a higher ratio of variable to constant loci with increasing GC content (Figure 3D).

Recovery of loci in all GBS methods is influenced by restriction site polymorphisms, which generate null alleles [33] or, more optimistically, presence-absence polymorphisms. With ddRAD-seq, null alleles are likely to be more frequent than in the original RAD-seq method because mutations at either recognition site can result in the gain or loss of a given locus. Null alleles result either in missing data, in the case of homozygous individuals, or heterozygous individuals erroneously scored as homozygotes. Given sufficient population sampling and sequencing depth, loci with null alleles at an appreciable frequency can be identified and removed from analyses; for such loci, observed genotypes will deviate from Hardy-Weinberg expectations, and sequencing depth for true homozygotes will be higher than for individuals with one copy of the null allele (Figure S6 in File S1). This approach, however, will not be effective for loci recovered at low depth. Likewise, substantial variation in depth across loci (see above) makes differences in average per-sample depth an ineffective tool for detecting loci with null alleles (see [16]). One conservative approach to reducing potential bias due to null alleles is to use only those loci recovered in all samples.

Gautier *et al.* [16] used simulations of RAD locus evolution to conclude that null alleles (or allele drop out, ADO) result in a counterintuitive upward bias on estimates of both genetic diversity and population divergence. Their simulation, however, modeled only the loss of existing RAD loci, such that restriction site mutations tended to reduce the observed frequency of ancestral (and more common) SNP states in the flanking sequence while increasing rare allele frequencies [16]. In real data, null alleles may be either ancestral or derived, so the generality of this finding is uncertain. As noted above, ddRAD loci provide a biased sample of the genome and will thus provide a biased estimate of genome-wide nucleotide diversity; as has long been standard procedure in molecular ecology, estimation of demographic and historical parameters will require calibration specifically for the loci under study.

The frequency of null alleles increases with population size and mutation rate (*θ* = 4*N_e_µ*) and, in comparisons between populations or species, with population divergence [16]. This limits the potential utility of restriction-enzyme-based methods for comparative or phylogenetic analyses involving highly divergent taxa, but it also presents an opportunity to use presence-absence data as a source of informative characters. Analysis of presence-absence data will require successful library preparation and good representation of loci across the selected size range. Two metrics that may be useful in evaluating the quality of library preparation when distantly related samples are included in the same study are: 1) within sample variation among loci in read depth for a core set of loci found in all samples, and 2) variation among samples in the total number of clusters (i.e., putative loci) with sequencing depth between appropriate minimum and maximum thresholds (scaled to the total number of reads for each sample). Samples with a smaller number of putative loci and/or greater variation in depth across loci are likely to be missing loci due to problems with fragment library preparation rather than natural variation.

In addition to restriction site polymorphisms, we note a number of other situations in which natural polymorphism generates either null alleles or variation in the length of alleles, and in turn variation in sequencing depth due to amplification bias. When using ddRAD-seq, an indel of sufficient length can move an allele out of the selected size range and generate a null allele even if both restriction sites are conserved. In other cases, both alleles are recovered but may differ substantially in length, and therefore sequencing depth. We also noted loci with polymorphisms in either SbfI or EcoRI sites, but alternative alleles that were nonetheless recovered due to nearby restriction sites that generated alleles of different length. A similar effect is observed in the original RAD-seq method, in which gain or loss of restriction sites over an ∼10 kb range influences the efficiency of hydroshearing and in turn allelic variation in sequencing depth [7].

### Computational challenges

While the intent of this study is not a thorough evaluation of the bioinformatics components of ddRAD-seq, we briefly note some issues that influence the extent to which homologous loci are recovered across a set of samples. RAD-seq reads may either be mapped to a reference genome or clustered *de* *novo* into putative loci based on an essentially arbitrary threshold of sequence similarity. A lower threshold may result in increased clustering of paralogous loci, whereas a higher threshold may result in failure to cluster divergent alleles. In initial analyses, we noted that alleles differing by a long indel often failed to cluster, an issue we addressed by merging clusters with identical or nearly identical BLAST hits. Given the likelihood of indels resulting from both sequencing error and natural polymorphism, multiple sequence alignment for each putative locus is an essential step in the process. Our current genotyping code also implements a gap-coding algorithm, so that each unique indel, regardless of length, is scored as a single presence-absence character. The computational approach we used for the analyses here is in other respects generally similar to that of Peterson *et al.* [6], including the retention of singleton sequences that may contain random errors but are nonetheless informative at genuinely polymorphic positions, and a focus on counting distinct haplotypes within each individual and comparing those counts with Mendelian expectations.

## Conclusions

Direct comparison of empirical ddRAD-seq data from a zebra finch sample with predictions from the reference genome reveals unexpected carryover of small fragments through the size selection process, amplification biases associated with fragment length and GC content, and overrepresentation of genomic regions with high GC content. These effects are consistent across samples, generating strongly correlated variation among samples in per locus sequencing depth. Preliminary data for indigobirds shows that our method recovers a large and broadly overlapping set of loci across individual samples and sequencing runs, generating sufficient sequence data to genotype 5,966 loci in all 46 samples and 9,833 loci in 42 of 46 samples (>90%), thresholds that are more stringent than applied in other recent studies seeking to recover robust genotypic data for most individuals (e.g., [6], [11], [34]). For the core set of 5,966 loci, median depth per sample per locus is sufficiently high that bias in estimating population genetic parameters such as the site frequency distribution should be minimal [35]. Given good success with ∼500 K sequence reads per sample, there is ample opportunity to increase the number of multiplexed samples using “combinatorial indexing” [6]. Thus, ddRAD-seq is an increasingly cost-effective approach for generating robust data for a sample of genomic loci and is well suited for those applications in molecular ecology not requiring dense sampling of the genome. Indeed, with increased multiplexing, ddRAD-seq will likely become an attractive and highly powerful replacement for microsatellite loci in paternity analyses, for example.

As noted above, we designed our method with the intention of generating robust genotypic data for a consistent set of loci across samples. Other GBS approaches yield data for a larger fraction of the genome and/or a larger number of individual samples, but with lower sequencing depth per locus. Indeed, it has recently been argued that sequencing at a depth of ∼1x per locus per individual and otherwise maximizing the number of individuals sampled is the optimal design for maximizing information about population genetic parameters using Bayesian approaches [36]. Pending further evaluation of this provocative result, the nearly complete data matrices produced by our method lend themselves to analysis with a broad range of existing population genetic models and software. At the same time, data generated by our method should be perfectly compatible with statistical approaches, including “direct estimation” of population genetic parameters from the data [35], which may yield additional information from those loci recovered at lower average depth. We also note that adding additional samples may represent a significant challenge for many studies of natural animal populations. Likewise, in other contexts in which there is limited sampling of divergent populations and/or closely related species, including phylogenetic applications, accurately inferring genotypes and allele frequencies based on incomplete information may be impracticable. In these situations, a method generating more robust data for individual samples and loci may be desirable.

Insights resulting from our study, including those related to amplification biases, biased genomic representation, size selection, star activity and null alleles, are also relevant for understanding potential biases in methods that target a larger number of loci at lower depth. Likewise, our laboratory protocol is easily modified to increase the number of sampled loci; replacing EcoRI with MseI increases the expected number of loci from ∼10 K to ∼100 k and replacing both enzymes with PstI and MseI increases the predicted number of loci to ∼1 million. All three combinations (SbfI-EcoRI, SbfI-MSeI, PstI-MseI) leave the same sticky ends, allowing a single set of bar-coded adapters to be used in a wide range of studies.

## Supporting Information

File S1 **Supporting figures and tables.** Table S1. Effect of restriction enzyme choice on number of ddRAD loci. Table S2. Sequences of adapters and PCR primers used in fragment library preparation. Table S3. Sequences for 128 6-bp barcodes. Figure S1. Sequencing depth and BLAST results for 17,144 “clusters” ( = putative loci) in the zebra finch empirical data. Figure S2. Bioanalyzer results comparing results of size selection with “full width” versus “tapered” gel cut. Figure S3. Performance of five indigobird ddRAD-seq runs in recovering a core set of loci. Figure S4. Correlation of sequencing depth between the reference sequencing run (RAD10) and subsequent runs using the same laboratory protocol. Figure S5. Variation among sequencing runs in number of loci recovered as a function of total read depth per sample. Figure S6. Per sample sequencing depth for a locus with a null allele.
(DOCX)

Protocol S1 **Outline of the “double-digest” restriction-site associated DNA sequencing (ddRAD-seq) protocol used in the study.**
(DOCX)
