# Supplementary material for: Amplification Biases and Consistent Recovery of Loci in a Double-Digest RAD-seq Protocol
Source: PLoS One. 2014 Sep 4;9(9):e106713. doi: 10.1371/journal.pone.0106713 (PMC4154734; doi:10.1371/journal.pone.0106713)
Supplement: File S1 — Supporting figures and tables. Table S1. Effect of restriction enzyme choice on number of ddRAD loci. Table S2. Sequences of adapters and PCR primers used in fragment library preparation. Table S3. Sequences for 128 6-bp barcodes. Figure S1. Sequencing depth and BLAST results for 17,144 “clusters” ( = putative loci) in the zebra finch empirical data. Figure S2. Bioanalyzer results comparing results of size selection with “full width” versus “tapered” gel cut. Figure S3. Performance of five indigobird ddRAD-seq runs in recovering a core set of loci. Figure S4. Correlation of sequencing depth between the reference sequencing run (RAD10) and subsequent runs using the same laboratory protocol. Figure S5. Variation among sequencing runs in number of loci recovered as a function of total read depth per sample. Figure S6. Per sample sequencing depth for a locus with a null allele. (DOCX) [file pone.0106713.s001.docx]

**Table S1. Effect of restriction enzyme choice on number of ddRAD loci.** Based on *in silico* digests of the zebra finch (*Taeniopygia* *guttata*) reference genome. Results assume SbfI as the primary enzyme (recognition sequence: CCTGCAGG) and a fragment size range of 200-400 bp.

| **Second Enzyme** | **Recognition Sequence** | **P1/P1 fragments** | **ddRAD loci** |
| --- | --- | --- | --- |
| SalI | G^TCGAC | 1,404 | 178 |
| AgeI | A^CCGGT | 1,399 | 561 |
| EagI | C^GGCCG | 1,380 | 1,040 |
| NheI | G^CTAGC | 1,393 | 1,595 |
| KpnI | GGTAC^C | 1,350 | 2,528 |
| MfeI | C^AATTG | 1,377 | 3,500 |
| SphI | GCATG^C | 1,303 | 4,007 |
| BamHI | G^GATCC | 1,273 | 5,494 |
| EcoRI | G^AATTC | 1,249 | 6,758 |
| SacI | GAGCT^C | 1,171 | 9,264 |
| NcoI | C^CATGG | 1,159 | 14,925 |

**Table S2. Sequences of adapters and PCR primers used in fragment library preparation.**

| Name | Sequence |
| --- | --- |
| P1.top | 5’-AATGATACGGCGACCACCGAGATCTACACTCTTTCCCTACACGACGCTCTTCCGATCTxxxxxxTGCA-3’ |
| P1.bottom | 5’-Phos-xxxxxxAGATCGGAAGAGCGTCGTGTAGGGAAAGAGTGTAGATCTCGGTGGTCGCCGTATCATT-3’ |
| P2.top* | 5’-Phos-AATTAGATCGGAAGAGCGGTTCAGCAGGAATGCCGAGACCGATCAGAACAA-3’ |
| P2.bottom* | 5’-CAAGCAGAAGACGGCATACGAGATCGGTCTCGGCATTCCTGCTGAACCGCTCTTCCGATCT-3’ |
| P2.top† | 5’-Phos-AATTAGATCGGAAGAGCACACGTCTGAACTCCAGTCACzzzzzzATCAGAACAA-3’ |
| P2.bottom† | 5’-CAAGCAGAAGACGGCATACGAGATzzzzzzGTGACTGGAGTTCAGACGTGTGCTCTTCCGATCT-3’ |
| RAD1.F | 5’-AATGATACGGCGACCACCGAG-3’ |
| RAD2.R | 5’-CAAGCAGAAGACGGCATACGAG-3’ |

*P2 adapter sequences used for RAD5 and RAD10; †P2 adapter sequences used for RAD14, 16, and 18; xxxxxx = barcode; Phos = phosphorylation; zzzzzz = index

**Table S3. Sequences for 128 6-bp barcodes.** All barcodes designed to have 50% GC content, no more than two consecutive identical bases, and at least two differences distinguishing all pairs of barcodes. Asterisks mark the subset of 48 barcodes used in this study.

| Barcode | Sequence |  | Barcode | Sequence |  | Barcode | Sequence |  | Barcode | Sequence |
| --- | --- | --- | --- | --- | --- | --- | --- | --- | --- | --- |
| **bc6_001** | **ACACCT*** |  | bc6_033 | CAACAG |  | bc6_065 | GAACAC |  | bc6_097 | TCACCA |
| bc6_002 | ACACGA |  | **bc6_034** | **CAACTC*** |  | **bc6_066** | **GAACTG*** |  | **bc6_098** | **TCACGT*** |
| **bc6_003** | **ACAGCA*** |  | bc6_035 | CAAGAC |  | bc6_067 | GAAGAG* |  | bc6_099 | TCAGCT |
| bc6_004 | ACAGGT |  | **bc6_036** | **CAAGTG*** |  | bc6_068 | GAAGTC |  | bc6_100 | TCAGGA |
| **bc6_005** | **ACCAAG*** |  | bc6_037 | CACACT |  | bc6_069 | GACACA |  | bc6_101 | TCCAAC |
| bc6_006 | ACCATC |  | bc6_038 | CACAGA |  | **bc6_070** | **GACAGT*** |  | **bc6_102** | **TCCATG*** |
| **bc6_007** | **ACCTAC*** |  | **bc6_039** | **CACTCA*** |  | bc6_071 | GACTCT |  | bc6_103 | TCCTAG |
| bc6_008 | ACCTTG |  | **bc6_040** | **CACTGT*** |  | **bc6_072** | **GACTGA*** |  | bc6_104 | TCCTTC |
| bc6_009 | ACGAAC |  | bc6_041 | CAGACA |  | **bc6_073** | **GAGACT*** |  | bc6_105 | TCGAAG |
| bc6_010 | ACGATG |  | bc6_042 | CAGAGT |  | bc6_074 | GAGAGA |  | **bc6_106** | **TCGATC*** |
| **bc6_011** | **ACGTAG*** |  | **bc6_043** | **CAGTCT*** |  | **bc6_075** | **GAGTCA*** |  | bc6_107 | TCGTAC |
| bc6_012 | ACGTTC |  | bc6_044 | CAGTGA |  | bc6_076 | GAGTGT |  | bc6_108 | TCGTTG |
| bc6_013 | ACTCCA |  | bc6_045 | CATCAC |  | **bc6_077** | **GATCAG*** |  | bc6_109 | TCTCCT |
| **bc6_014** | **ACTCGT*** |  | **bc6_046** | **CATCTG*** |  | **bc6_078** | **GATCTC*** |  | **bc6_110** | **TCTCGA*** |
| bc6_015 | ACTGCT |  | bc6_047 | CATGAG |  | bc6_079 | GATGAC |  | bc6_111 | TCTGCA |
| bc6_016 | ACTGGA |  | bc6_048 | CATGTC |  | bc6_080 | GATGTG |  | bc6_112 | TCTGGT |
| **bc6_017** | **AGACCA*** |  | bc6_049 | CTACAC |  | bc6_081 | GTACAG |  | bc6_113 | TGACCT |
| **bc6_018** | **AGACGT*** |  | bc6_050 | CTACTG |  | **bc6_082** | **GTACTC*** |  | bc6_114 | TGACGA |
| bc6_019 | AGAGCT |  | **bc6_051** | **CTAGAG*** |  | bc6_083 | GTAGAC |  | bc6_115 | TGAGCA |
| bc6_020 | AGAGGA |  | **bc6_052** | **CTAGTC*** |  | bc6_084 | GTAGTG |  | **bc6_116** | **TGAGGT*** |
| bc6_021 | AGCAAC |  | **bc6_053** | **CTCACA*** |  | bc6_085 | GTCACT |  | **bc6_117** | **TGCAAG*** |
| **bc6_022** | **AGCATG*** |  | **bc6_054** | **CTCAGT*** |  | **bc6_086** | **GTCAGA*** |  | bc6_118 | TGCATC |
| bc6_023 | AGCTAG |  | bc6_055 | CTCTCT |  | bc6_087 | GTCTCA |  | bc6_119 | TGCTAC |
| **bc6_024** | **AGCTTC*** |  | bc6_056 | CTCTGA |  | bc6_088 | GTCTGT |  | bc6_120 | TGCTTG |
| bc6_025 | AGGAAG |  | bc6_057 | CTGACT |  | bc6_089 | GTGACA |  | bc6_121 | TGGAAC |
| bc6_026 | AGGATC |  | bc6_058 | CTGAGA |  | **bc6_090** | **GTGAGT*** |  | **bc6_122** | **TGGATG*** |
| **bc6_027** | **AGGTAC*** |  | **bc6_059** | **CTGTCA*** |  | bc6_091 | GTGTCT |  | bc6_123 | TGGTAG |
| **bc6_028** | **AGGTTG*** |  | **bc6_060** | **CTGTGT*** |  | bc6_092 | GTGTGA |  | bc6_124 | TGGTTC |
| **bc6_029** | **AGTCCT*** |  | **bc6_061** | **CTTCAG*** |  | bc6_093 | GTTCAC |  | **bc6_125** | **TGTCCA*** |
| **bc6_030** | **AGTCGA*** |  | **bc6_062** | **CTTCTC*** |  | **bc6_094** | **GTTCTG*** |  | bc6_126 | TGTCGT |
| bc6_031 | AGTGCA |  | bc6_063 | CTTGAC |  | bc6_095 | GTTGAG |  | bc6_127 | TGTGCT |
| bc6_032 | AGTGGT |  | bc6_064 | CTTGTG |  | bc6_096 | GTTGTC |  | bc6_128 | TGTGGA |

**Figure S1.** **Sequencing depth and BLAST results for 17,144 “clusters” (= putative loci) in the zebra finch empirical data.** BLAST results for clusters in each of five sequencing depth categories are shown along with the number of clusters in each category.

**
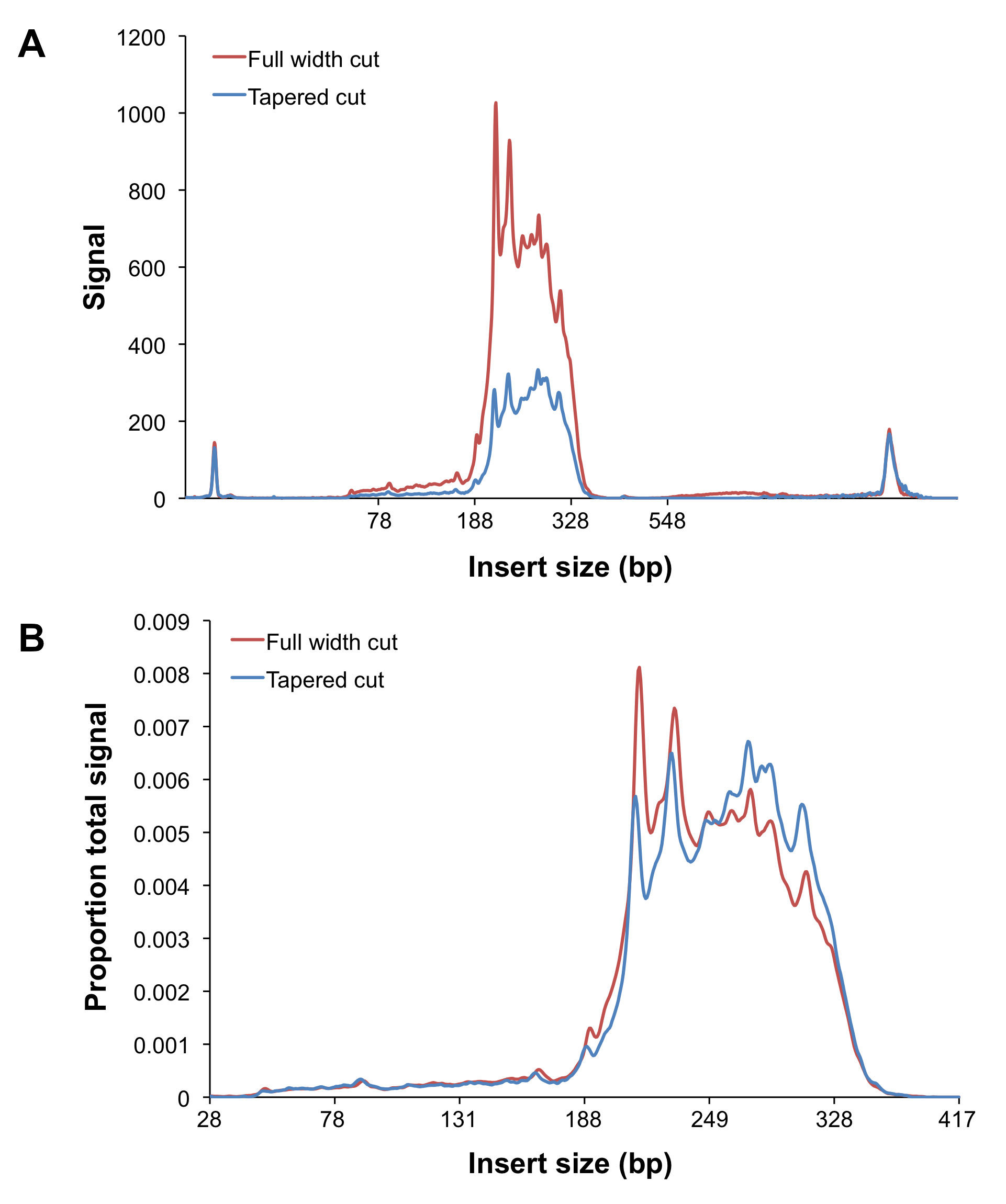
**

**Figure S2. Bioanalyzer results comparing results of size selection with “full width” versus “tapered” gel cut.** A single sample was split into equal volumes following the digestion and ligation steps and loaded into separate gel lanes. One lane was size selected by cutting the full width of the lane from 300 to 450 bp (178-328 bp exclusive of adapters), while the other lane was excised using a tapered cut, going from full width at 450 bp to half the width of the lane at 300 bp. (A) Overlaid bioanalyzer results for PCR products from the full width and tapered cut lanes. Cutting the full width of the lane resulted in more template DNA being added to the PCR and higher PCR product concentrations. The small fragment carry carryover effect (i.e., fragments smaller than the size range being selected) is evident at insert sizes ~40-180 bp. (B) Proportion of total signal versus size (shown for the relevant size range). The tapered cut resulted in proportionally less signal for insert sizes ~180-250 bp, and proportionally more signal for insert sizes ~250-330 bp compared to the full width cut.

**
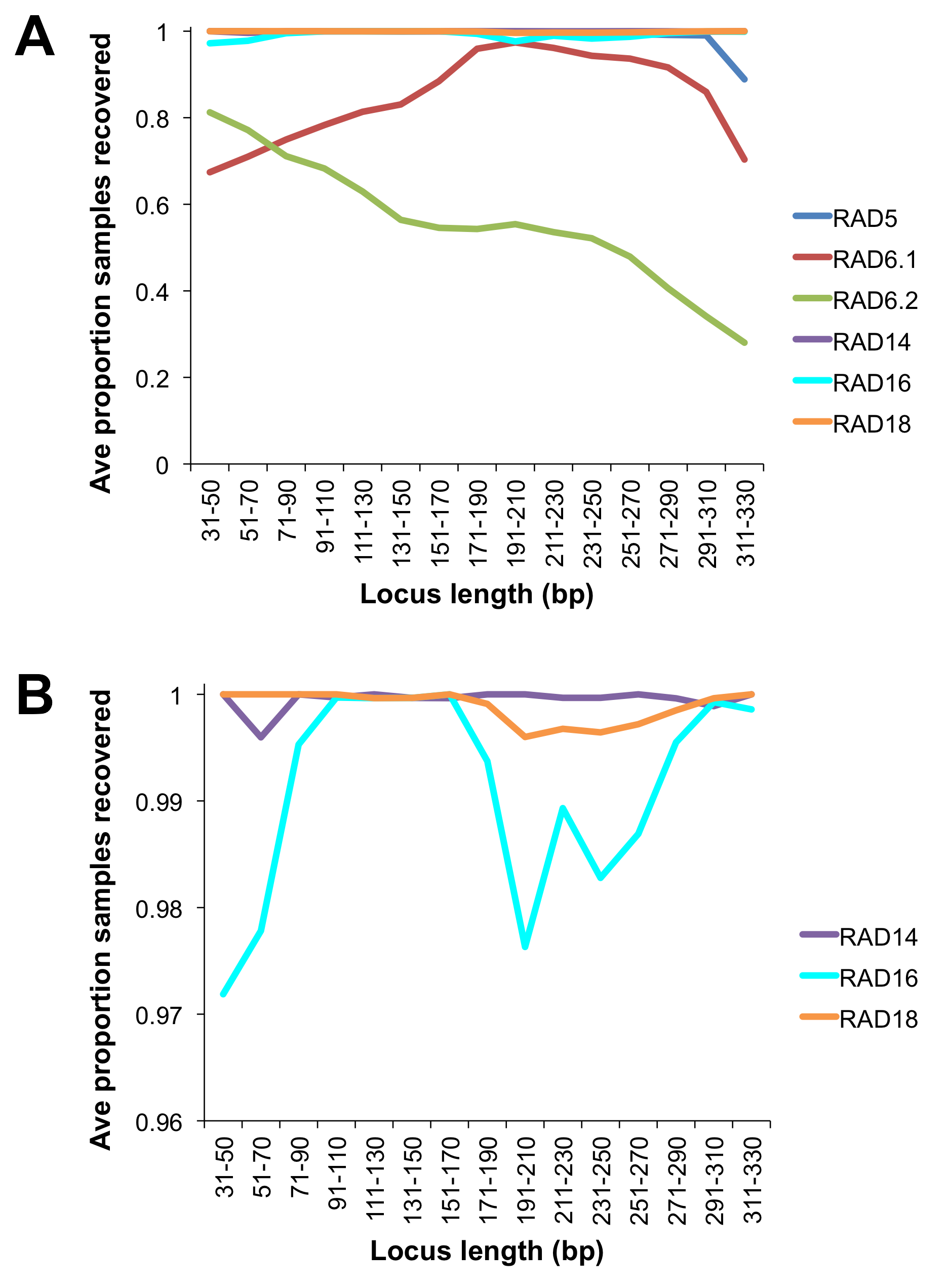
**

**Figure S3. Performance of five indigobird ddRAD-seq runs in recovering a core set of loci.** The core set comprises 5,996 loci that were genotyped in all 46 samples in RAD10 with a depth of at least five sequence reads per sample per locus. (A) The average proportion of samples with data (sequencing depth ≥1) in each run as a function of locus length (*n* = 10 samples per run). The length of each locus was determined either directly from the empirical data (for loci shorter than the read length) or was estimated from BLAST results against the zebra finch genome. Predicted lengths are subject to some error (see text) and were not available for 1,661 of these loci. (B) Same as panel (A), but showing results only for RAD14, 16, and 18 and the upper range of the y-axis.


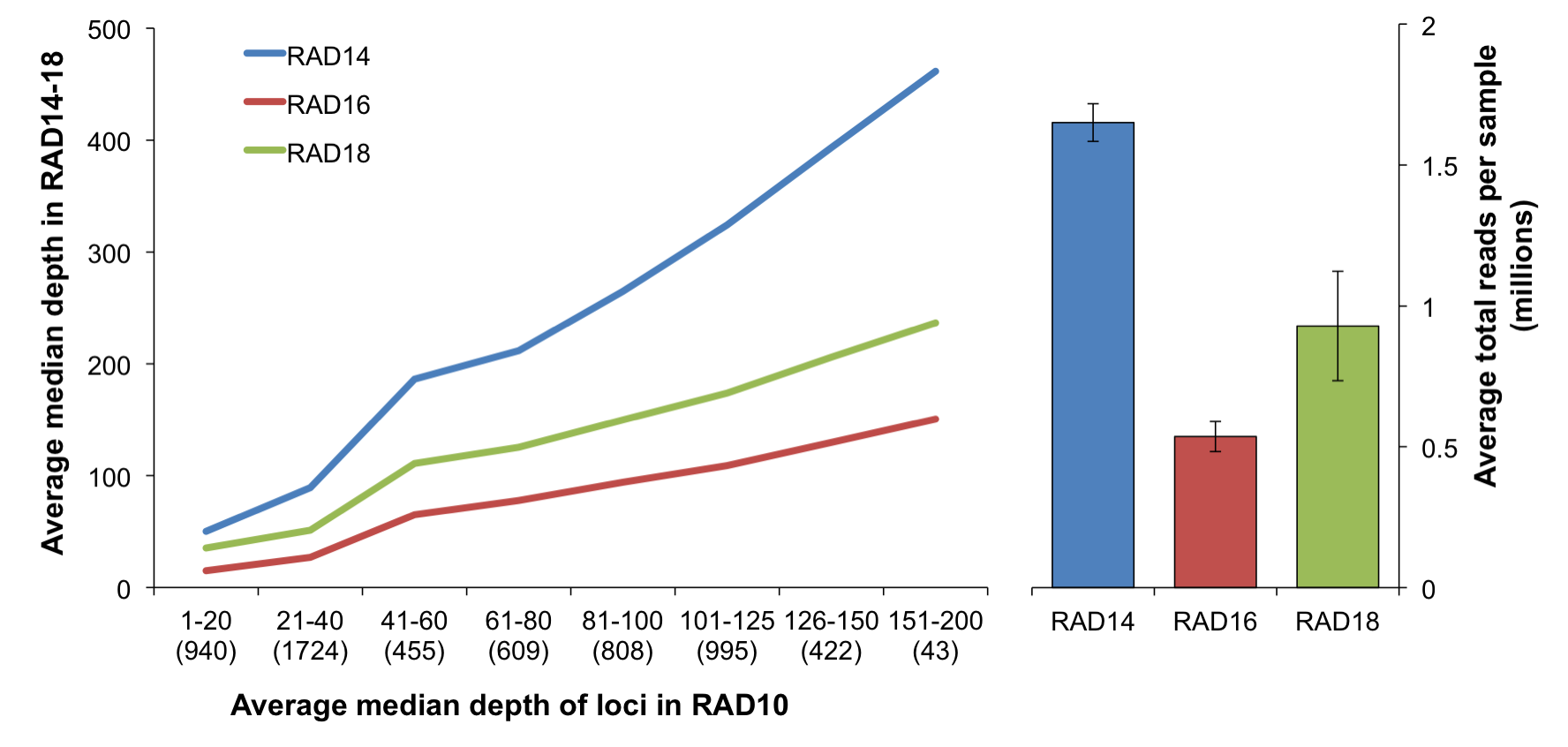


**Figure S4. Correlation of sequencing depth between the reference sequencing run (RAD10) and subsequent runs using the same laboratory protocol.** Based on a core set of 5,996 loci that were genotyped in all 46 samples in RAD10 with a depth of at least five reads per sample per locus. Numbers in parentheses below the x-axis show the number of loci in each sequencing depth bin. Bar graphs show the average (± sd) number of reads assigned to each sample analyzed in subsequent runs (*n* = 10 samples per run). Note that higher overall sequencing depth in RAD14 results in a proportionate increase in depth across all loci, with “high depth” loci accumulating additional reads more quickly than “low depth” loci.


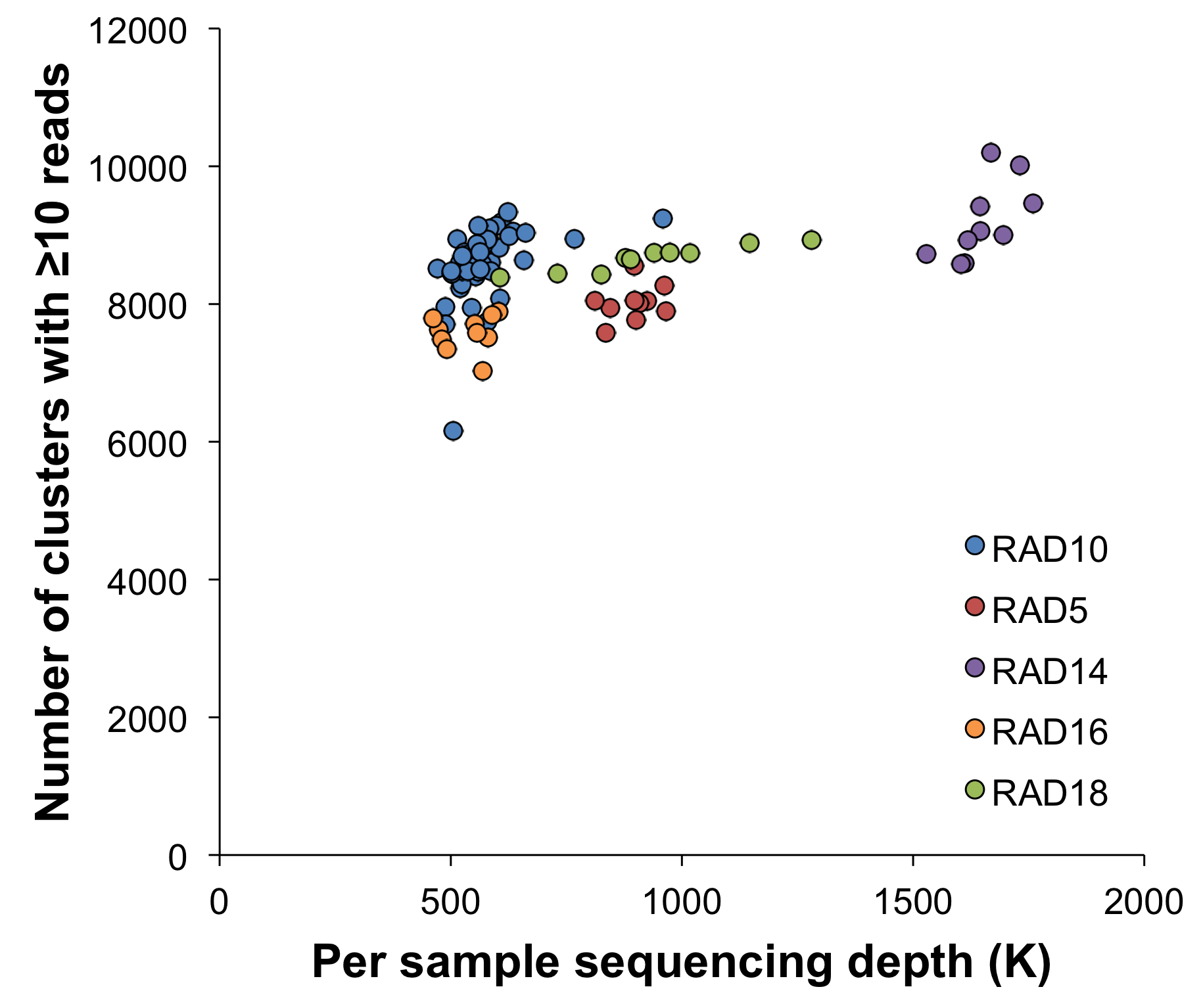


**Figure S5. Variation among sequencing runs in number of loci recovered as a function of total read depth per sample.**


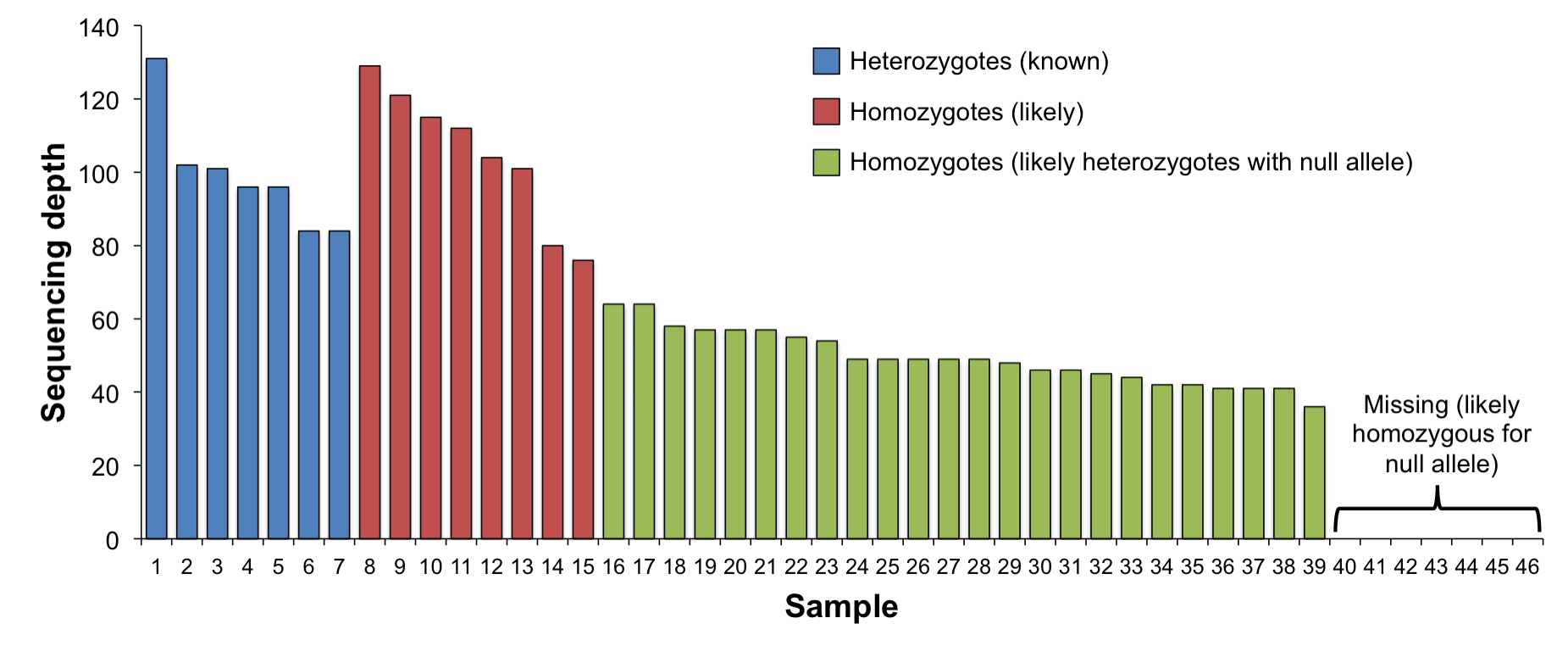


**Figure S6. Per sample sequencing depth for a locus with a null allele.** Despite relatively high depth per sample, there are seven samples with zero reads. If we assume that these samples are homozygous for a null allele then the population frequency of the null allele is ~0.39 (square root of 7/46), and the frequency of all other alleles is ~0.61. The expected frequency of heterozygotes with one null allele (which will appear to be a homozygotes) is 0.48 (2*0.39*0.61). We therefore expect ~22 (48%) of the 46 samples to be false homozygotes (i.e., heterozygotes with the null allele) and to have lower sequencing depth than other samples. The green bars indicate the 24 samples with the lowest sequencing depth, all of which are lower than any of the known heterozygotes (blue). Homozygotes with relatively high sequencing depth (red) comparable to known heterozygotes are likely to be homozygous for the sequenced allele.
