# Supplementary material for: Amplification Biases and Consistent Recovery of Loci in a Double-Digest RAD-seq Protocol
Source: PLoS One. 2014 Sep 4;9(9):e106713. doi: 10.1371/journal.pone.0106713 (PMC4154734; doi:10.1371/journal.pone.0106713)
Supplement: Protocol S1 — Outline of the “double-digest” restriction-site associated DNA sequencing (ddRAD-seq) protocol used in the study. (DOCX) [file pone.0106713.s002.docx]

**Boston University ddRAD-seq laboratory protocol**

**Jeffrey M. DaCosta & Michael D. Sorenson**

**Revised: 4 April 2014**

**Overview of ddRAD-seq protocol**

1. Double-digest genomic DNA with two restriction enzymes that both produce “sticky ends” with two different overhangs.
2. Ligate adapters to digested DNA.
   1. Barcoded P1 adapter ligates to overhang produced by enzyme with fewer cut sites.
   2. Divergent-Y P2 adapter ligates to overhang produced by enzyme that cuts more frequently.
3. *Size select samples in agarose gel. Purify with Qiagen MinElute kit.
4. Amplify size-selected fragments via Phusion PCR.
5. Purify PCR products with AMPure XP beads.
6. Quantify purified PCR products with qPCR.
7. Pool samples in equimolar amounts.
8. Evaluate library concentration and quality, submit for Illumina sequencing.

*NOTE: This protocol can be streamlined by pooling samples after ligation and prior to size-selection. Briefly, when using this strategy, we employ the following changes to the protocol:

*After ligation and before size selection:*

- Quantify adapter-ligated fragments with KAPA qPCR kit (use 1:10 dilution of samples).
- Create sub-pools of 12 samples in equimolar amounts. For each sub-pool, use the entire available volume of the sample with the lowest concentration in order to maximize sub-pool molarity (see step 7).

*Size selection:*

- Sub-pools are typically >300 µL in volume, and thus are split into multiple lanes (75-100 µL per lane) during gel electrophoresis.
- Gel-extract each lane (with wedge-cut) using the Qiagen MinElute kit (i.e., there are multiple extractions for each sub-pool).

*Amplification:*

- For each gel extraction product, amplify fragments as described in step 4 (i.e., there are multiple amplification reactions for each sub-pool).

*PCR purification:*

- Purify products of each reaction using SPRI beads (see step 5). Combine multiple elutions from the same sub-pool into a single tube.

*Sub-pool quantification and pooling:*

- Estimate sub-pool concentrations with qPCR (see step 6). Identify the sub-pool with the fewest total nanomoles (noting that sub-pools can be different volumes), and then pool the sub-pools in equimolar amounts as described in step 7.

**1. Double-digest genomic DNA (gDNA)**

**Materials:**

- High quality genomic DNA (we use the Qiagen DNeasy Blood & Tissue Kit for DNA extraction)
- SbfI-HF restriction enzyme (R3642L, NEB)
- EcoRI-HF restriction enzyme (R3101S, NEB)
- 10x NEBuffer 4 (B7004S, NEB)

**Procedure:**

1. Clean work area before and after procedure. Use aerosol tips when pipetting.
2. Estimate concentration of gDNA extracts using Nanodrop or Qubit. We also recommend that extract quality be evaluated on an agarose gel; degraded DNA will produce a smear of low molecular weight fragments.
3. Each digestion reaction is 50 µL and contains the following mix of reagents:

Genomic DNA ~1 µg (volume varies depending on DNA concentration)

*EcoR*I-HF 1 µL (20 U)

*Sbf*I-HF 1 µL (20 U)

10x NEBuffer 4 5 µL

Water as needed to bring total volume to 50 µL

*Note: reaction volume can be increased if necessary for a low concentration extract*.

1. We recommend running a positive control to ensure that the reaction is successful. We use a mitochondrial DNA PCR product that includes recognition sites for both restriction enzymes. A successful digest cuts this product into three bands.
2. Create a master mix of the reagents common to each reaction, multiplying volumes below by number of samples:

| Reagent | µL/rxn |
| --- | --- |
| *EcoR*I-HF | 1 |
| *Sbf*I-HF | 1 |
| NEBuffer 4 | 5 |

1. Add 7 µL of the master mix to each tube.
2. Add DNA extract and water to each tube in appropriate volumes.
3. Complete digest in thermocycler.

30 min digest at 37°C

20 min enzyme deactivation at 65°C

Slow cooling to room temperature, hold at 4°C

1. Run half of the positive control (i.e., 25 µL) in an agarose check gel to ensure that digestion was successful.

**2. Ligate Illumina Adapters**

**Materials:**

- Adapters (see Appendix I for preparation instructions):
  - 50 nM P1 adapters (use a different barcoded adapter for each sample)
  - 50 nM P2 paired-end compatible adapter (same for each sample in batch)
- 10x NEBuffer 2 (B7002S, NEB)
- T4 DNA Ligase (M0202T, NEB)
- rATP (E6011, Promega)

**Procedure:**

1. Clean work area before and after procedure. Use aerosol tips for all pipetting operations.
2. Each ligation reaction is 70 µL and contains the following mix of reagents:

Digested DNA sample 50 µL (full volume of digestion reaction)

10x NEBuffer 2 2 µL

rATP 0.6 µL

P1 adapter (50 nM) 4 µL

P2 adapter (50 nM) 12 µL

Water 0.4 µL

T4 ligase (add last) 1 µL

*Proportions of P1 and P2 adapters may need to be optimized for expected proportions of R1-R2, R1-R1, and R2-R2 restriction fragments based on the enzymes used and genome size and GC content.*

1. Perform ligation on the remaining volume of the positive control. Reduce the volume of all reagents by half (i.e., final volume of 35 µL rather than 70 µL) for the positive control sample.
2. Create a master mix of the reagents common to each reaction (except ligase), multiplying volumes below by number of samples:

| Reagent | µL/rxn |
| --- | --- |
| 10X NEBuffer 2 | 2 |
| rATP | 0.6 |
| P2 adapter | 12 |
| Water | 0.4 |

1. Add 15 µL of the master mix to each tube.
2. Add 4 µL of the appropriate barcoded P1 adapter to each tube. Pipette mix sample when adding the adapter to ensure that the solution is homogenous before adding ligase.
3. Add 1 µL of T4 ligase to each tube. As T4 ligase is active at room temperature, it should be the last reagent added to a well-mixed solution. Cover tubes and vortex.
4. Complete ligation in thermocycler.

30 min ligation at 22°C

20 min enzyme deactivation at 65°C

Slow cooling to room temperature, hold at 4°C

**3. Size Selection with Gel Electrophoresis**

*Alternatives: Pippin Prep, E-Gel SizeSelect, SPRI beads*

**Materials:**

- Low melt agarose (e.g. GeneMate Sieve GQA, ISC Bioexpress)
- Custom internal size standards (300 and 450 bp – see Appendix II or create your own PCR products of the appropriate sizes)
- Ethidium bromide (10 mg/ml), or appropriate staining substitute
- Lithium borate (LB) buffer (10mM lithium hydroxide monohydrate + 30mM boric acid, pH ~8.5)
- MinElute Gel Extraction Kit (28604, Qiagen)

**Procedure:**

1. Clean work area before and after procedure. Wash gel rigs and combs before using. Use aerosol tips when pipetting.
2. Prepare 2% low melt agarose in LB buffer. When cool, but still liquid, spike with 1 µL ethidium bromide per 40 ml of agarose. Pour a thick gel with wide wells in order to accommodate at least 70 µL of sample in each lane. Allow the gel to cool for at least 1 hour (note that low melt agarose gels take longer to set up as compared to standard agarose gels).
3. Submerse gel in LB buffer spiked with ethidium bromide (1 µL per 40 ml of buffer).
4. Add 4 µL of the pooled internal size standards (300 and 450 bp) to each sample (the full 70 µL volume from ligation reaction).
5. Load samples and the positive control.
6. Electrophorese gel for ~165 minutes at 100 volts.
7.
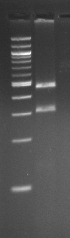
Cut each sample out of the gel individually using a “tapered cut”

The downstream PCR reaction preferentially amplifies smaller fragments. Making a tapered cut reduces the proportion of smaller fragments, which helps to reduce the effect of this bias. Execute size selection by cutting on the internal edge of each size standard. Excise the space between the size standards such that the entire width of the lane at the high end is captured, but only half of the width of the lane at the low end of the size range (see red outline in figure to right). Transfer each gel slice to one or more labeled microcentrifuge tubes. Wash the scalpel with water between each tapered cut.

*If the size of the excised gel necessitates using more than one tube per sample, cut excised gel slice into multiple vertical sections so that each tube contains the full size range.*

1. Cut the appropriate band/restriction fragment from the lane containing the positive control (i.e., the restriction fragment with a different sticky end on either side, which should now be ligated to P1 and P2 adapters on either end).

*Use the MinElute kit to extract DNA from the gel slices for each sample and the positive control.*

1. Weigh the gel slices. Add 3 volumes of Buffer QG for every 1 volume of gel volume (100 mg ≈ 100 µL).
2. Incubate at room temperature until the gel slice is completely dissolved. Mix frequently by vortexing.

*Note that the Qiagen protocol calls for incubation at 50°C, but dissolving the gel at room temperature improved the recovery of AT-rich fragments (see Quail et al. 2008, Nat. Meth.).*

1. After the gel slice is dissolved, check that the Buffer QG is still yellow.
2. Add 1x gel volume of isopropanol to each sample/tube. Invert tubes several times to mix solution.
3. Transfer up to 750 µL of each sample to a labeled MinElute column in a collection tube. Centrifuge for 1 min at 10,000 rpm and discard flow-through. This step will need to be repeated in order to transfer the complete volume for each sample through the column.
4. Add 500 µL of Buffer QG. Spin for 1 min at 10,000 rpm. Discard flow-through.
5. Add 750 µL of Buffer PE. Incubate at room temp for 2-4 minutes.
6. Spin for 1 min at 10,000 rpm. Discard flow-through.
7. Spin for an additional 1 min at 10,000 rpm.
8. Transfer column to a labeled microcentrifuge tube.
9. Add 20 µL of Buffer EB. Incubate at room temperature for at least 1 min. Spin for 1 min at 10,000 rpm.

*Note that the Qiagen protocol calls 10 µL of Buffer EB (following Etter et al. 2011. Mol. Meth. Evol. Gen.*)*.*

**4. PCR amplification of size-selected DNA**

**Materials:**

- Amplification primers (10 µM)

RAD1.F: 5′-AATGATACGGCGACCACCGAG-3′

RAD2.R: 5′-CAAGCAGAAGACGGCATACGAG-3′

- Phusion High-Fidelity PCR Master Mix (F-531S, Finnzymes)

**Procedure:**

1. Clean work area before and after procedure. Use aerosol tips when pipetting.
2. Each PCR reaction is 60 µL and contains the following mix of reagents:

| **Reagent** | **μL/reaction** |
| --- | --- |
| Phusion Mix | 30 |
| Water | 9 |
| F primer | 3 |
| R primer | 3 |
| Template DNA | 15 |

1. Be sure to include both a positive and negative (water) control when calculating volumes for the master mix.
2. Create a master mix of the reagents common to each reaction, multiplying volumes below by number of samples:

| **Reagent** | **µL/rxn** |
| --- | --- |
| Phusion Mix | 30 |
| Water | 9 |
| F primer | 3 |
| R primer | 3 |

1. Add 45 µL of master mix to each tube.
2. Add 15 µL of the appropriate template DNA (purified, size-selected ligation product) to each tube.
3. Amplify in thermocycler with the following profile:

30 sec at 98°C

20 cycles of the following:

10 sec at 98°C

30 sec at 60°C

40 sec at 72°C

5 min at 72°C, hold at 4°C

1. Run the positive control in a gel to confirm that Phusion PCR was successful.

**5. Purify PCR products with magnetic SPRI beads**

**Materials:**

- AMPure XP SPRI beads (Agencourt) or homebrew mixture (see *Rohland & Reich 2012, Gen. Res*.)
- Super Magnetic plate (A32782, Agencourt)
- 70% ethanol (made fresh from 100% ethanol and reagent grade water)
- Reagent grade water or Qiagen Buffer EB

**Procedure:**

1. Clean work area before and after procedure. Use aerosol tips when pipetting.
2. Gently shake the bottle holding the magnetic bead solution to ensure a homogeneous solution.
3. Add 108 µL of magnetic bead solution to each sample (i.e., 1.8x PCR reaction volume).
4. Mix beads and PCR products by pipette mixing 10 times. Let the mixed samples incubate at room temperature for 5 minutes. *Pipette mixing is preferable, as it tends to be more reproducible. The color of the mixture should be homogeneous after mixing.*
5. Place the microtubes onto the magnetic plate. Incubate for 2 minutes at room temperature to separate the beads from the solution. Wait for mixture to become clear before proceeding to the next step.
6. Do NOT remove the samples from the magnetic plate. Aspirate the supernatant from each sample and discard. Do not disturb the ring of magnetic beads.
7. Do NOT remove the samples from the magnetic plate. Add 200 µL of fresh 70% ethanol to each sample. Incubate for 30 seconds at room temperature. Aspirate out the ethanol and discard.
8. Repeat step #7.
9. Aspirate again with fine pipette tips to ensure that all ethanol has been removed. Allow microtubes to dry for 10 minutes at room temperature.
10. Remove microtubes from the magnetic plate. Add 40 µL of reagent grade water or elution buffer (Buffer EB from MinElute kit) to each sample and pipette mix 10 times. *With a 40 µL elution volume, the liquid level will be high enough to contact the magnetic beads. If less volume is used, then extra pipette mixing is needed to ensure that the liquid comes into contact with the beads.*
11. Place the microtubes back onto the magnetic plate. Wait 1 minute for the beads to separate from the solution.
12. Transfer the eluant for each sample to a labeled 1.5 mL centrifuge tube.

**6. Quantify purified PCR products with qPCR**

**Materials:**

- 384-well plate
- Clear adhesive plate cover
- Library quantification kit (KK4832, KAPA Biosystems)
- Sample dilution buffer: 10 mM Tris, pH 8.0 + 0.05% Tween 20

Recipe for 500 mL:

0.6 g Tris

~490 mL nanopure water

250 µL Tween 20

Adjust pH and bring solution to 500 mL

**Procedure:**

1. Clean work area before and after procedure. Use aerosol tips when pipetting.
2. Dilute individual fragment libraries 1000-fold. Combine 999 µL of dilution buffer with 1 µL of each library. Vortex.
3. Repeat step 2 two more times to produce three independent, 1000x dilutions of each library.
4. Prepare SYBR master mix as follows, multiplying the volumes below by number of samples plus controls and standards:

| **Reagent** | **μL/reaction** |
| --- | --- |
| SYBR FAST mix + primers | 6 |
| Water | 2 |

*Make sure that the primer mix has been added to the SYBR solution. Note that the six standards, a no-template control, and your samples are all run in triplicate. Also, it is usually necessary to add an extra 10% to the number of reactions/wells to allow for some pipetting loss.*

1. Dispense 8 µL of the master mix to the appropriate wells of a 384-well plate.
2. Add 2 µL of the kit standards, diluted sample libraries, or dilution buffer (no template control) to appropriate wells in the 384-well plate.
3. Spin plate and ensure that there are no bubbles at the bottom of wells and check for any debris on the outside/bottom of the plate. Cover plate with a clear adhesive film and run the assay on the qPCR machine following KAPA instructions.

**7-8. Pool samples in equimolar amounts and quality control**

**Procedure:**

1. For the sample with the lowest DNA concentration (C_low_), calculate the number of nmoles (X_low_) in the sample. The volume of the aliquot should be ~37 µL (40 µL elution from bead cleanup minus 3 µL used in qPCR), but assume 35 µL. X_low_ represents the maximum amount of DNA (nmoles) that you can get from this sample, and thus sets the upper limit of nmoles/sample.

X_low_ = (V*C_low_)/1,000,000

*Note: the 1,000,000 is to adjust the units for volume from L to µL*

Example: C_low_ = 3 nM (i.e., lowest qPCR result for a batch of samples that you want to pool)

V = 35 µL

X_low_ = (35*3)/1,000,000 = 0.000105 nmoles

1. Calculate the necessary volume of the other samples (V_i_) in the pool using the following equation:

V_i_ = (X_low_/C_i_)*1,000,000

*where X_low_ = number of nmoles for sample with lowest concentration*

*C_i_ = concentration (nM) of sample i*

Example: X_low_ = 0.000105 nmoles

C_i_ = 6 nM

V_i_ = (0.000105/6)*1,000,000 = 17.5 µL

1. The final concentration of the pool will equal the harmonic mean of the individual sample concentrations in the mixture.
2. Pool samples, taking an appropriate volume from each based on the calculations above. Use the same P10 pipetter for all pipetting operations (drawing 10 µL with a P10 three times will be more accurate than drawing 30 µL with a P200 once).
3. Ensure that calculations and pooling were executed correctly by verifying the concentration of the pooled library with an additional qPCR assay.
4. Evaluate the size distribution of DNA fragments in the pooled library with a Bioanalyzer (Agilent Technologies) or Fragment Analyzer (Advanced Analytical) assay.
5. In our experience, qPCR and Bioanalyzer/Fragment Analyzer assays often generate conflicting concentration estimates, which make it difficult to calculate optimal library loading parameters. Although results may vary among sequencing facilities, we have achieved favorable cluster densities and data quality in HiSeq 2000 and HiSeq 2500 runs using TruSeq v3 reagents, a library concentration estimate derived from a Fragment Analyzer, and a loading concentration of 6 pM that includes a 10% spike of PhiX DNA to improve basecalling through low complexity bases (i.e. barcode and restriction site sequences).

**Appendix I – Preparation of adapters:**

**Materials:**

P1 Adapter:

Top: 5′-AATGATACGGCGACCACCGAGATCTACACTCTTTCCCTACACGACGCTCTTCCGATCTxxxxxxTGCA-3′

Bottom: 3′-TTACTATGCCGCTGGTGGCTCTAGATGTGAGAAAGGGATGTGCTGCGAGAAGGCTAGAxxxxxx-Phos-5′

P2 Adapter (compatible with single-end or paired-end run):

Top: 5′-Phos-AATTAGATCGGAAGAGCACACGTCTGAACTCCAGTCACzzzzzzATCAGAACAA-3′

Bottom: 3′-TCTAGCCTTCTCGTGTGCAGACTTGAGGTCAGTGzzzzzzTAGAGCATACGGCAGAAGACGAAC-5′

xxxxxx = barcode

zzzzzz = index

Red: sticky end overhang

Blue: “divergent-Y”

- 10x Annealing buffer (500 mM NaCl, 100 mM Tris-Cl, pH 7.5-8.0)

**Procedure:**

1. Order oligos with different barcodes (P1) and indices (P2). Phosphates should be added to the 5′ end of the adapter strands that will be ligated to the DNA fragment.
2. Reconstitute oligos and mix in equimolar amounts with final concentration of annealing buffer at 1x.

Example:

Reconstitute

| **Oligo** | **Conc. (nm)** | **Water added (µL)** | **Final Con. (µM)** |
| --- | --- | --- | --- |
| Top | 31.46 | 283.14 | 111.1 |
| Bottom | 30.51 | 274.59 | 111.1 |

Mix for annealing

| **Reagent** | **Volume (µL)** |
| --- | --- |
| 10x annealing buffer | 10 (add first) |
| Top strand | 45 |
| Bottom strand | 45 |

*Produces double-stranded adapter at a final concentration of 50 µM.*

1. Heat samples on heat block at 95°C for 5 min. Turn off heat block and allow the block to slowly cool to room temperature (~4 hours).
2. Dilute adapters to 50 nM.
   1. Combine 100 µL of 10x annealing buffer with 899 µL of water. Vortex.
   2. Add 1 µL of 50 µM adapter. Vortex.

**Appendix II – Preparation of internal size standards:**

**Materials:**

- Internal size standards are amplified from redhead duck (*Aythya* *americana*) genomic DNA using two mtDNA primer sets:

300 bp standard:

RH300.F 5′-TGAGTAACTTGGGGCCACATC-3′

RH300.R 5′-TGATTGCGCTACCTTTGCAC-3′

450 bp standard:

RH450.F 5′-CACAAGATGCACCTAAACACACC-3′

RH450.R 5′-CTGCTAAATCCGCCTTCCAG-3′

- dNTP mix
- 10X PCR buffer
- MgCl_2_ solution (25 mM)
- AmpliTaq Gold DNA polymerase (Applied Biosystems)

**Procedure:**

1. Clean work area before and after procedure. Use aerosol tips when pipetting.
2. To produce 120 µL of a size standard, set up four 30 µL reactions with the following mix of reagents:

| **Reagent** | **μL/reaction** |
| --- | --- |
| Water | 16 |
| 10X buffer | 3 |
| MgCl_2_ | 3 |
| dNTP mix | 3 |
| F primer | 1.5 |
| R primer | 1.5 |
| AmpliTaq Gold | 0.15 |
| Template DNA | 2 |

1. Amplify in thermocycler (touchdown protocol 58-52°C).
2. Run small volume of each reaction and a negative control in an agarose check gel confirm that PCR was successful. *Amplified products from two size standards can be pooled into a single tube.*
